# The effect of HLA genotype on disease onset and severity in CTLA-4 insufficiency

**DOI:** 10.3389/fimmu.2024.1447995

**Published:** 2025-01-06

**Authors:** Sara Posadas-Cantera, Noriko Mitsuiki, Florian Emmerich, Virginia Patiño, Hanns-Martin Lorenz, Olaf Neth, Ingunn Dybedal, Kjetil Taskén, Alejandro A. Schäffer, Bodo Grimbacher, Laura Gámez-Díaz

**Affiliations:** ^1^ Institute for Immunodeficiency, Center for Chronic Immunodeficiency (CCI), Medical Center, Faculty of Medicine, University of Freiburg, Freiburg, Germany; ^2^ Institute of Medical Microbiology and Hygiene, Medical Center– University of Freiburg, Faculty of Medicine, University of Freiburg, Freiburg, Germany; ^3^ Institute for Transfusion Medicine and Gene Therapy, Freiburg University Medical Center, Faculty of Medicine, University of Freiburg, Freiburg, Germany; ^4^ Immunology Team, American Insurance, Montevideo, Uruguay; ^5^ Division of Rheumatology, Department of Internal Medicine V, University of Heidelberg, Heidelberg, Germany; ^6^ Paediatric Infectious Diseases, Rheumatology and Immunology Unit, Hospital Universitario Virgen del Rocío, Instituto de Biomedicina de Sevilla, IBiS/Universidad de Sevilla/CSIC, Seville, Spain; ^7^ Department of Hematology and Pharmacology, Oslo University Hospital, Oslo, Norway; ^8^ Department of Cancer Immunology, Institute for Cancer Research, Oslo University Hospital and Institute of Clinical Medicine, University of Oslo, Oslo, Norway; ^9^ Cancer Data Science Laboratory, National Cancer Institute, National Institutes of Health, Bethesda, MD, United States; ^10^ Department of Rheumatology and Clinical Immunology, Medical Center- University of Freiburg, Faculty of Medicine, University of Freiburg, Freiburg, Germany; ^11^ CIBSS– Centre for Integrative Biological Signalling Studies, University of Freiburg, Freiburg, Germany; ^12^ RESIST– Cluster of Excellence 2155 to Hannover Medical School, Satellite Center Freiburg, Freiburg, Germany

**Keywords:** cytotoxic T-lymphocyte antigen 4 (CTLA-4), immune dysregulation, inborn errors of immunity (IEI), disease modifiers, human leukocyte antigen (HLA), genetic linkage analysis

## Abstract

**Introduction:**

Human Cytotoxic-T-lymphocyte-antigen-4 (CTLA-4) insufficiency caused by heterozygous germline mutations in *CTLA4* is a complex immune dysregulation and immunodeficiency syndrome presenting with reduced penetrance and variable disease expressivity, suggesting the presence of disease modifiers that trigger the disease onset and severity. Various genetic and non-genetic potential triggers have been analyzed in CTLA-4 insufficiency cohorts, however, none of them have revealed a clear association to the disease. Multiple HLA haplotypes have been positively or negatively associated with various autoimmune diseases and inborn errors of immunity (IEI) due to the relevance of MHC in the strength of the T cell responses.

**Methods:**

In this exploratory study, we investigated the association of disease onset, severity and clinical manifestations of CTLA-4 insufficiency with specific HLA class I (A, B and C) and class II (DRB1 and DQB1) alleles in forty-three individuals harboring heterozygous mutations in *CTLA4*. Twenty-six out of the 43 recruited individuals presented moderate or severe clinical symptoms whereas 17 were completely healthy. HLA frequency analysis, odds ratio analysis and genetic linkage analysis were used.

**Results:**

The principal statistical analyses showed no positive association between the HLA genotypes analyzed with the disease onset or the disease severity. We found potential risk associations of HLA-DQB1*05:01 and HLA-DRB1*01:02 with respiratory tract involvement and HLA-C*05:01 with affection of the neurological system in the CTLA-4-insufficient patients. Additionally, we found a potential protective association of HLA-DRB1*01:01 with gastrointestinal symptoms.

**Discussion:**

Even though, our findings suggest that HLA-A, -B, -C, DRB1, and DQB1 do not contribute to the onset or severity of disease in CTLA-4 insufficiency, certain HLA-alleles may influence the manifestation of specific symptoms. We advocate for further investigation of specific class I and class II HLA alleles as potential disease modifiers in larger clinical cohorts of CTLA-4 insufficiency.

## 1 Introduction

The complete activation of T lymphocytes requires at least two signals. The first signal involves the recognition of the major histocompatibility complex (MHC)-bound peptide on antigen presenting cells (APC) by the T-cell receptor (TCR) on T lymphocytes, determining the strength of the T-cell response (1). The second signal involves the binding of B7 costimulatory molecules on APCs with the CD28 receptor expressed on T lymphocytes. Following T-cell activation, intracellular vesicles containing cytotoxic-T-lymphocyte-antigen-4 (CTLA-4) are mobilized to the cell membrane to outcompete CD28 for binding to B7 costimulatory molecules on APCs. After binding, CTLA-4:B7 complexes are internalized into T lymphocytes via transendocytosis for further lysosomal degradation (2). Hence, CTLA-4 is considered a negative immune regulator essential for controlling T-cell activation and maintaining immune homeostasis.

Heterozygous germline mutations in *CTLA4* have been identified as the cause of an inborn error of immunity (IEI) known since 2015 as CTLA-4 insufficiency (3, 4). This syndrome is characterized by immunodeficiency and immune dysregulation, leading to clinical symptoms such as hypogammaglobulinemia, recurrent infections, autoimmune cytopenia, hepatosplenomegaly, tissue lymphoid infiltrations, and malignancies (3–6). However, CTLA-4 insufficiency presents with incomplete penetrance and reduced disease expressivity as about 30% of *CTLA4* mutation carriers exhibit no clinical symptoms despite having impaired *in vitro* CTLA-4 function (3, 7). These observations suggest the presence of disease modifiers that trigger disease onset or influence disease severity. To date, several genetic and non-genetic disease modifiers in the context of CTLA-4 insufficiency have been studied. Among non-genetic factors, past infections by Epstein Barr virus (EBV), cytomegalovirus, herpes simplex virus type 1 and 2 and *Toxoplasma gondii* have been ruled out (8). Regarding genetic factors, a gain-of-function mutation in *JAK3* and a loss-of-function mutation in *CLEC7A* have been proposed as disease modifiers as they seem to contribute to the full disease expressivity in patients with heterozygous mutations in *CTLA4* (9, 10). However, these two rare variants in *JAK3* and *CLEC7A* have been identified in only one patient each.

The MHC system is required to deliver the first signal of T cell activation; MHC is also known as the human leukocyte antigen (HLA) system in humans and contains the most polymorphic gene cluster of the entire human genome. Based on the structure and function of the HLA gene products, HLA components are classified into three main groups, class I, class II and class III (11). HLA class I, which includes *HLA-A, HLA-B* and *HLA-C* isotypes, presents endogenous peptides to the TCR of CD8^+^ T cells. In contrast, the TCR of CD4^+^ T cells recognize exogenous peptides bound to HLA class II that includes *HLA-DR, DP*, and *DQ* isotypes. HLA class III encodes, among others, components of the complement system, heat shock proteins and tumor necrosis factor family proteins (12, 13). Specific HLA haplotypes have been commonly associated with disease predisposition or protection in various autoimmune diseases. For instance, several *HLA-DRB1* alleles have been associated with susceptibility to rheumatoid arthritis (RA) in different populations, *HLA-DQA1* and *HLA-DQB1* alleles have been strongly associated with type-1 diabetes and celiac disease, and specific HLA-DRB3*03:01 allele with Crohn’s disease (14, 15). In addition, susceptibility to common variable immunodeficiency (CVID) and deficiency of IgA (sIgAD), both IEI, has also been found to be positively associated with *HLA-DQ* and *HLA-DRB1* alleles (16, 17). These associations are not surprising, given that the specificity of the HLA-peptide–TCR (T-cell receptor) interaction is essential for maintaining an efficient and appropriate adaptive immune response, not only to counteract infection and malignancy, but also to preserve self- tolerance, preventing the generation of autoimmune diseases. The specific underlying mechanisms behind these associations are largely unknown.

We hypothesized that specific HLA alleles may influence disease manifestation, disease onset and disease severity in CTLA-4 insufficiency. In this exploratory study, we analyzed the A, B and C HLA class I alleles, and the DRB1 and DQB1 class II HLA alleles in 43 individuals from seven different families carrying heterozygous mutations in *CTLA4.* Our results indicate that the investigated HLA alleles are unlikely to be disease modifiers in patients with CTLA-4 insufficiency.

## 2 Materials and methods

### 2.1 Patient characteristics

Between 2018 and 2022, we enrolled 43 individuals from seven different families, each harboring heterozygous mutations in *CTLA4.* These families are identified here as families A, B, C, D, E, F and G. Families A, B and E are from Germany and were recruited at the Center for Chronic Immunodeficiencies, Medical Center - University of Freiburg. Family C was referred from the Department of Immunology and Histocompatibility, Centre for Primary Immunodeficiencies, “Aghia Sophia” Children’s Hospital, Athens, Greece, whereas Family D was referred from the Immunology Team, Montevideo, Uruguay. Families F and G attend the Paediatric Infectious Diseases, Rheumatology and Immunology Unit, Hospital Virgen del Rocio, Instituto de Biomedicina de Sevilla, Spain, and the Department of Hematology, Oslo University Hospital, Norway, respectively. All participating individuals donated blood samples for DNA extraction after signing an informed written consent under local ethics board–approved protocols from each of the above-mentioned institutions. This project was approved by the local ethics committee of University of Freiburg, protocol number 466/18.

### 2.2 HLA genotyping

Genomic DNA samples were extracted from either whole blood or peripheral blood mononuclear cells following standard protocols. HLA typing encompassing HLA-A, -B, -C, -DRB1, and -DQB1 was performed applying routine methods either through Sanger-Sequencing (ProTrans, Hockenheim, Germany) or next generation sequencing (NGS) using NGSgo^R^ sequencing kits (GenDx, the Netherlands). HLA-typing analysis was performed by aligning the nucleotide sequences to the official HLA-reference database (https://www.ebi.ac.uk/ipd/imgt/hla/), which is used as the reference for research and clinical diagnostics worldwide.

### 2.3 Single HLA allele frequency analysis

Allele-specific frequencies were calculated in cases and controls irrespective of their pedigree status. All HLA alleles were coded as either present or absent.

### 2.4 Genetic linkage analysis of the HLA locus on chromosome 6

We used FASTLINK v. 4.1P (18–20) to compute logarithm of odds (LOD) scores for the observed data and we used the related simulation package FASTSLINK v. 3.0 (21) to estimate the power to detect linkage. For simplicity, we show single-marker analyses with four different parameter settings, while noting that other parameter settings gave qualitatively similar results. We modeled the modifier locus as dominant because there are parent-child affected pairs. We assumed a penetrance of 0.9 (90%) for the modifier disease associated allele. In three out of four parameter combinations, we allowed a phenocopy rate of 0.01, meaning that the modifier would not be active if there is a phenocopy. We varied the frequency of the modifier allele among (0.01, 0.10, 0.20, 0.50). The genetic linkage analysis differs fundamentally from the HLA allele frequency analysis in that the LOD scores for each family are computed separately for each family and added. Thus, the disease-associated marker allele(s) can be different in each family and one can still detect genetic linkage. We assumed that the frequency of each HLA allele is equal for each locus. For the power calculations we used a 5-allele marker placed at 5 centiMorgans from the putative modifier locus. Using 100 replicates for each parameter setting, we estimated the probability of observing a LOD score above the thresholds of 1, 2, or 3.

### 2.5 CTLA-4 haploinsufficiency-morbidity score

The CHAI-morbidity score is a disease assessment score that was developed based on the data of the first 133 CTLA-4 insufficiency patients analyzed (7) and an additional 73 unpublished patients. The CHAI morbidity score quantifies organ involvement by integrating specific laboratory values including flow cytometry results, imaging data, and physiological functional results into a score and is described in detail in (22).

### 2.6 Statistical analysis

Pie charts were generated using GraphPad Prism version 9.0.0, Odds ratios and 95% confidence intervals were calculated using the R package epitools and visualized with the ggplot2 R package.

## 3 Results

### 3.1 Study population

**Figure 1:**
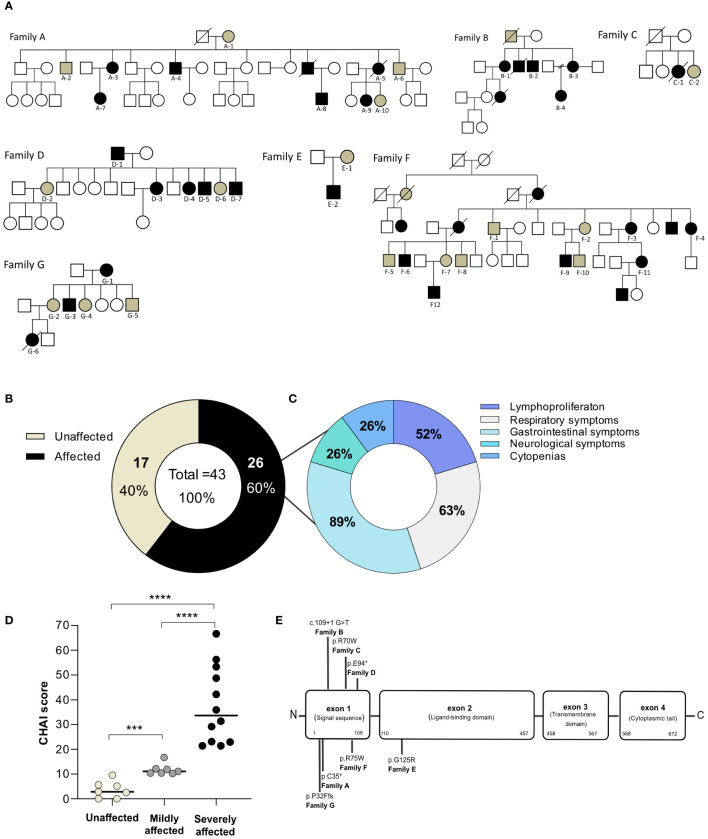
Characteristics of the study population. **(A)** Family pedigrees depicting *CTLA4* mutation carriers and recruited individuals. Squares indicate male subjects and circles indicate female subjects. Filled symbols indicate subjects with an identified mutation in *CTLA4*. Black symbols designate affected patients whereas brown symbols designate unaffected *CTLA4* mutation carriers. Recruited individuals are designated with an ID below their corresponding symbol. Crossed-out symbols indicate deceased subjects. **(B)** Classification of *CTLA4* mutation carriers into unaffected (brown) and affected groups (black) based on the requirement of medical attention. **(C)** Clinical symptoms of the affected *CTLA4* mutation carriers clustered in five main groups according to the organ affected. **(D)** CHAI score classified the *CTLA4* mutation carriers into unaffected <10 points (light brown), mildly affected between 10 to 20 points (gray) and severely affected >20 points (black) (n=19). Mann Whitney test. p-value >0.0001. **(E)** Schematic representation of the type and distribution of the *CTLA4* mutations identified in our cohort. *p<0.05; **p<0.01; ***p<0.001, ****p<0.0001.

We collected data from seven families, including a total of 43 individuals carrying heterozygous mutations in *CTLA4* (**Figure 1A**). Six of these families were from European countries, and one family was from South America. Eighteen out of the 43 recruited individuals were male (41.8%) while 25 were female (58.2%). At recruitment, the average age of our carrier cohort was 45.8 years, with an age range from 15 to 91 years (**Tables 1**, **2**).

**Table 1:** Baseline characteristics of the study population.

Family code	Nationality	Gender	Age	Affected vs Unaffected status	CHAI-Score (%)	Disease severity
A1	Germany	F	91	unaffected	unknown	n.a.
A2		M	64	unaffected	unknown	n.a.
A3		F	63	affected	10.42	mildly affected
A4		M	60	affected	35.9	severely affected
A5		F	dead	affected	16.67	mildly affected
A6		M	53	unaffected	unknown	n.a.
A7		F	32	affected	66.67	severely affected
A8		M	24	affected	56.25	severely affected
A9		F	27	affected	31.37	severely affected
A10		F	24	unaffected	0	n.a.
B1	Germany	F	61	affected	10.42	mildly affected
B2		M	55	affected	53.33	severely affected
B3		F	48	affected	29.17	severely affected
B4		F	21	affected	11.11	mildly affected
C1	Greece	F	dead	affected	unknown	unknown
C2		F	17	unaffected	unknown	n.a.
D1	Uruguay	M	68	affected	unknown	unknown
D2		F	29	unaffected	unknown	n.a.
D3		F	36	affected	unknown	unknown
D4		F	32	affected	unknown	unknown
D5		M	30	affected	unknown	unknown
D6		F	20	unaffected	unknown	n.a.
D7		M	24	affected	unknown	unknown
E1	Germany	F	53	unaffected	unknown	n.a.
E2		M	26	affected	48.72	severely affected
F1	Spain	M	72	unaffected	5.12	n.a.
F2		F	69	unaffected	9.5	n.a.
F3		F	66	affected	11.9	mildly affected
F4		F	56	affected	21.4	severely affected
F5		M	50	unaffected	2.56	n.a.
F6		M	46	affected	21.4	severely affected
F7		F	45	unaffected	0	n.a.
F8		M	37	unaffected	5.6	n.a.
F9		M	45	affected	12.12	mildly affected
F10		M	42	unaffected	2.8	n.a.
F11		F	40	affected	23.07	severely affected
F12		M	15	affected	10.2	mildly affected
G1	Norway	F	85	affected	unknown	unknown
G2		F	54	unaffected	unknown	n.a.
G3		M	53	affected	22.92	severely affected
G4		F	50	unaffected	unknown	n.a.
G5		M	48	unaffected	unknown	n.a.
G6		F	dead	affected	42.22	severely affected

n.a., not applicable.

**Table 2:** Clinical characteristics of the study population.

Characteristics	Affected		Unaffected	
	n=26	%	n=17	%
Gender (female)	15	57.9%	10	58.8%
Current age (mean +SD)*	44.0 + 18.3		48.1 + 19.5	
Non-malignant lymphoproliferation				
Lymphadenopathy	10	38.5%	0	0%
Splenomegaly	12	46.2%	0	0%
Hepatomegaly	5	19.2%	0	0%
Organ lymphocyte infiltrations**	12	46.2%	0	0%
Respiratory tract abnormalities				
Respiratory infections	14	53.8%	1	5.9%
Bronchiectasis	4	15.4%	0	0%
GLILD	8	30.8%	0	0%
Gastrointestinal symptoms				
Diarrhea/enteropathy	16	61.5%	2	11.8%
Atrophic gastritis	3	11.5%	1	5.9%
Celiac disease	1	3.8%	0	0%
Cytopenias				
Autoimmune hemolytic anemia (AIHA)	2	7.7%	0	0%
Idiopathic thrombocytopenia purpura (ITP)	6	23.1%	0	0%
Neutropenia	1	3.8%	0	0%
Pure red cell aplasia (PRCA)	2	7.7%	0	0%
Skin manifestations				
Psoriasis	11	42.3%	1	5.9%
Eczema	4	15.4%	0	0%
Other	7	26.9%	0	0%
Infectious microorganisms				
Epstein Barr Virus (EBV)	5	19.2%	0	0%
Recurrent herpes virus simplex I	4	15.4%	0	0%
Cytomegalovirus (CMV)	1	3.8%	0	0%
Other viruses	3	19%	1	6.25%
Salmonella spp.	4	15.4%	0	0%
Other bacteria	6	23.1%	0	0%
Candida spp.	7	26.9%	0	0%
Aspergillus spp.	2	7.7%	0	0%
Other Clinical symptoms				
Sepsis	5	19.2%	0	0%
Arthritis	6	23.1%	0	0%
Diabetes type 1	1	3.8%	0	0%
Hypothyroidism	4	15.4%	2	12.5%
CNS involvement	6	23.9%	1	5.9%
Malignancy	4	15.4%	2	12.5%
Hypo/Disgammaglobulinemia	18	81.8%	3	25%

*Age at the time of death for three patients.

**Lung, bone marrow, liver and kidney were the most affected organs for lymphocyte infiltration.

Based on the clinical status information provided by the treating physicians or extracted from the literature (7, 22, 23), the 43 recruited individuals were classified as *affected* or *unaffected CTLA4* mutation carriers (**Table 1**). The *affected* group included all *CTLA4* mutation carriers experiencing moderate or severe clinical symptoms, whereas the *unaffected* group included all those *CTLA4* mutation carriers not requiring specialized medical treatment up to the time of this study (7, 23). Out of the 43 *CTLA4* mutation carriers, 26 were classified as *affected* and 17 were classified as *unaffected* (**Figure 1B** and **Table 1**). Gender distribution was conserved in the *affected* (11 male and 15 female) and the *unaffected* groups (7 male and 10 female). The average age was also similar in the *affected* (44 years old) and *unaffected* groups (48.1 years old) (**Table 2**). At the time of the study, 40 patients were alive, while three *affected CTLA4* mutation carriers had passed away at the ages of 20 (code A5), 25 (code C1), and 37 (code G6) years.

### 3.2 Clinical and genetic characterization

Clinical manifestations of the 26 *affected CTLA4* mutation carriers were clustered into five main different groups: i) non-malignant lymphoproliferation, ii) respiratory tract illnesses, iii) gastrointestinal symptoms, iv) neurological clinical manifestations, v) cytopenias (**Figure 1C**). Non-malignant lymphoproliferation included lymphadenopathy, splenomegaly, hepatomegaly, and lymphocyte organ infiltrations. Respiratory tract conditions included respiratory infections, bronchiectasis and Granulomatous and Lymphocytic Interstitial Lung Diseases (GLILD). Gastrointestinal symptoms included diarrhea, enteropathy and atrophic gastritis. The cytopenias group involved autoimmune hemolytic anemia (AIHA), idiopathic thrombocytopenia purpura (ITP), neutropenia and pure red cell aplasia (PRCA). Clinical manifestations are summarized in **Table 1**. Based on the CHAI morbidity score calculated in 19 *affected* individuals, twelve patients were classified as severely affected (CHAI score >20%), and seven as mildly affected (score between 10% and 20%) (**Figure 1D** and **Table 1**).

Sequencing of *CTLA4* confirmed, two nonsense mutations, three missense mutations, one insertion and one frameshift mutation. Specifically, Family A and Family D had nonsense mutations (c.105C>A; p.C35*, and c.280G>T; E94*, respectively), whereas Family C, E and F had missense mutations (c.208C>T; p.R70W, c.373G>A; p.G125R, and c.223C>T; p.R75W, respectively). Family B had one insertion (c.109 + 1G>T), and one frameshift mutation was found in Family G (c.94_101delinsTTCTCTTCATCA; p.P32Ffs*) (**Figure 1E**). Functional testing of CTLA-4-mediated transendocytosis in regulatory T cells from all members of Families A (n=10), B (n=4), C (n=2) and F (n=12) reported elsewhere, showed reduced transendocytosis of CD80 regardless the *affected* or *unaffected* status of the individual (3, 7).

### 3.3 HLA genotyping

HLA genotyping of HLA-A,-B,-C was performed in 38 *CTLA4* mutation carriers due to sample availability whereas 43 *CTLA4* mutation carriers were sequenced for -DRB1 and -DQB1 alleles (**Table 2**). The genotype information included a two-to-four digit HLA allele. For each *clinical manifestation group* (**Figure 1C**), allele-specific overall frequencies were calculated in cases and controls irrespective of the pedigree status. We predicted the haplotypes of G-1, G-2, and G-4 based on allele frequencies. The genotype of HLA-DRB1 of G-1, G-2, and G-4 presented up to two-digits as DRB1*13s. In addition, the most likely genotype formed a haplotype with DQB1*06:04, which has been reported as DRB1*13:02 in the allele frequency net database (AFND) (24). The results of genotypes and serotypes are shown in **Table 3**.

**Table 3:** HLA haplotype associations.

Patient	Genotype				
	*HLA-A*	*HLA-B*	*HLA-C*	*HLA-DRB1*	*HLA-DQB1*
A-1	*03:01, *24:02	*35:01, *35:03	*04:01	*11:01, *13:01	*03:01, *06:03
A-2	*03:01, *24:02	*07:02, *35:03	*04:01, *07:02	*08:01, *13:01	*04:02, *06:03
A-3	*03:01	*07:02, *35:01	*04:01, *07:02	*08:01, *11:01	*03:01, *04:02
A-4	*24:02, *68:01	*15:01, *35:03	*03:04, *04:01	*10:01, *13:01	*05:01, *06:03
A-5	*03:01, *24:02	*07:02, *35:03	*04:01, *07:02	*08:01, *13:01	*04:02, *06:03
A-6	*24:02, *68:01	*15:01, *35:03	*03:04, *04:01	*10:01, *13:01	*05:01, *06:03
A-7	*03:01, *29:02	*08:01, *35:01	*04:01, *07:01	*07:01, *11:01	*02:02, *03:01
A-8	*02:01, *24:02	*07:02, *35:03	*04:01, *07:02	*13:01, *15:01	*06:02, *06:03
A-9	*01:01, *03:01	*07:02, *08:01	*07:02	*03:01, *08:01	*02:01, *04:02
A-10	*01:01, *03:01	*07:02, *08:01	*07:02	*03:01, *08:01	*02:01, *04:02
B-1	*01:01, *03:01	*07:02	*07:02	*13:01, *15:01	*06:02, *06:03
B-2	*01:01	*07:02, *08:01	*07:01, *07:02	*03:01, *15:01	*02:01, *06:02
B-3	*01:01, *03:01	*07:02	*07:02	*13:01, *15:01	*06:02, *06:03
B-4	*03:01, *03:20	*07:02, *51:08	*07:02, *16:02	*07:01, *13:01	*02:02, *06:03
C-1	*02:01, *11:01	*35:01, *39:06	*04:01, *12:03	*01:01, *16:01	*05:01, *05:02
C-2	*02:01, *24:02	*39:06, *51:01	*01:02, *12:03	*04:03, *16:01	*03:02, *05:02
D-1	*02:01, *24:02	*35:01, *51:01	*04:01, *14:02	*01:02, *08:01	*04:02, *05:01
D-2	*02:01	*44:03, *51:01	*14:02, *16:01	*01:02, *13:01	*05:01, *06:03
D-3	*02:01, *24:02	*35:01, *44:03	*04:01, *16:01	*08:01, *13:01	*04:02, *06:03
D-4	*01:01, *02:01	*51:01, *57:01	*07:01, *14:02	*01:02, *07:01	*03:03, *05:01
D-5	*02:01	*44:03, *51:01	*14:02, *16:01	*01:02, *13:01	*05:01, *06:03
D-6	*01:01, *02:01	*51:01, *57:01	*07:01, *14:02	*01:02, *07:01	*03:03, *05:01
D-7	*01:01, *02:01	*51:01, *57:01	*07:01, *14:02	*01:02, *07:01	*03:03, *05:01
E-1	*23:01, *24:02	*07:02, *15:71	*03:03, *07:02	*01:01, *11:01	*03:01, *05:01
E-2	*24:02, *30:01	*07:02, *35:02	*04:01, *07:02	*01:01, *15:01	*05:01, *06:02
F-1	*24:02, *30:02	*38:01, *44:03	*04:01, *12:03	*07:01, *13:01	*02:02, *06:03
F-2	*24:02, *30:02	*38:01, *44:03	*04:01, *12:03	*07:01, *13:01	*02:02, *06:03
F-3	*02:01, *68:02	*35:02, *35:08	*04:01	*03:01, *11:01	*02:01, *03:01
F-4	*02:01, *24:02	*35:08, *38:01	*04:01, *12:03	*03:01, *13:01	*02:01, *06:03
F-5	*01:01, *68:02	*35:02, *52:01	*04:01, *12:02	*11:01, *15:02	*03:01, *06:01
F-6	*01:01, *68:02	*35:02, *52:01	*04:01, *12:02	*11:01, *15:02	*03:01, *06:01
F-7	*02:01, *11:01	*18:01, *35:08	*04:01, *12:03	*03:01, *11:04	*02:01, *03:01
F-8	*01:01, *02:01	*35:08, *52:01	*04:01, *12:02	*03:01, *15:02	*02:01, *06:01
F-9	*26:01, *30:02	*27:02, *44:03	*02:02, *04:01	*07:01, *11:01	*02:02, *03:01
F-10	*26:01, *30:02	*27:02, *44:03	*02:02, *04:01	*07:01, *11:01	*02:02, *03:01
F-11	*02:01, *03:01	*35:08, *52:01	*04:01, *12:02	*03:01, *15:01	*02:01, *05:01
F-12	*11:01	*18:01	*05:01, *12:03	*03:01, *11:04	*02:01, *03:01
G-1	n.a.	n.a.	n.a.	*13:02+, *04:04	*03:02, *06:04
G-2	n.a.	n.a.	n.a.	*13:02+, *15:01	*06:02, *06:04
G-3	n.a.	n.a.	n.a.	*04:01, *04:04	*03:01, *03:02
G-4	n.a.	n.a.	n.a.	*13:02+, *15:01	*06:02, *06:04
G-5	n.a.	n.a.	n.a.	*04:04, *15:01	*03:02, *06:02
G-6	*02:01	*08:01, *44:02	*05:01, *07:01	*03:01, *15:01	*02:01, *06:02

n.a, not available.

### 3.4 Single HLA allele frequencies association with risk of CTLA-4 insufficiency onset, severity or organ involvement

The odds ratio (OR) is widely used to quantify the association between an outcome and exposure to a factor. OR >1 indicates higher odds in the exposed individuals, implying increased risk of the outcome, while an OR <1 suggests lower odds, indicating protection. The 95% confidence interval (95% CI) estimates the precision of the OR and is often used as an indicator of statistical significance when it does not overlap the null value (OR= 1) (25). To further assess the potential association of single HLA allele frequencies with disease onset, severity or symptoms in CTLA-4 insufficiency, OR were derived from contingency tables, 95% CI were obtained with the adjusted Wald method for small sample size. Contingency tables containing zero values were adjusted using the Haldane and Anscombe correction (26). P-values were calculated using Fisher’s exact test without adjustment for multiple testing due to the exploratory nature of the study. However, Bonferroni-adjusted p-values are displayed in the association tables (**Table 4**; **Supplementary Tables 1**-**6**).

**Table 4:** Association of HLA-I and HLA-II alleles with affected status of *CTLA4* mutation carriers.

Allele	Affected	Unaffected	Odds.Ratio	p.value	p.adjusted (Bonferroni)	Lower.CI	Upper.CI
*HLA-A*	n=24	n=14					
*01:01	7	4	1.03	1.00	1	0.25	4.04
*02:01	11	5	1.52	0.74	1	0.40	5.46
*03:01	8	3	1.83	0.49	1	0.40	7.25
*03:20	1	0	1.85	0.53	1	0.10	15.01
*11:01	2	1	1.18	1.00	1	0.12	8.45
*23:01	0	1	0.18	0.15	1	0.02	3.37
*24:02	7	7	0.41	0.30	1	0.11	1.61
*26:01	1	1	0.57	1.00	1	0.05	6.08
*29:02	1	0	1.85	0.53	1	0.10	15.01
*30:01	1	0	1.85	0.53	1	0.10	15.01
*30:02	1	3	0.16	0.13	1	0.03	1.61
*68:01	1	1	0.57	1.00	1	0.05	6.08
*68:02	2	1	1.18	1.00	1	0.12	8.45
*HLA-B*	n=24	n=14					
*07:02	9	3	2.20	0.47	1	0.48	8.53
*08:01	4	1	2.60	0.63	1	0.28	14.19
*15:01	1	1	0.57	1.00	1	0.05	6.08
*15:71	0	1	0.18	0.15	1	0.02	3.37
*18:01	1	1	0.57	1.00	1	0.05	6.08
*27:02	1	1	0.57	1.00	1	0.05	6.08
*35:01	5	1	3.42	0.38	1	0.37	17.56
*35:02	3	1	1.86	1.00	1	0.19	11.16
*35:03	3	3	0.52	0.65	1	0.10	2.77
*35:08	3	2	0.86	1.00	1	0.14	4.76
*38:01	1	2	0.26	0.54	1	0.04	2.71
*39:06	1	1	0.57	1.00	1	0.05	6.08
*44:02	1	0	1.85	0.53	1	0.10	15.01
*44:03	3	4	0.36	0.39	1	0.08	1.84
*51:01	4	3	0.73	1.00	1	0.15	3.48
*51:08	1	0	1.85	0.53	1	0.10	15.01
*52:01	2	2	0.55	0.62	1	0.08	3.66
*57:01	2	1	1.18	1.00	1	0.12	8.45
*HLA-C*	n=24	n=14					
*01:02	0	1	0.18	0.15	1	0.02	3.37
*02:02	1	1	0.57	1.00	1	0.05	6.08
*03:03	0	1	0.18	0.15	1	0.02	3.37
*03:04	1	1	0.57	1.00	1	0.05	6.08
*04:01	14	9	0.78	1.00	1	0.21	2.98
*05:01	2	0	3.22	0.52	1	0.19	20.61
*07:01	5	1	3.42	0.38	1	0.37	17.56
*07:02	9	3	2.20	0.47	1	0.48	8.53
*12:02	2	2	0.55	0.62	1	0.08	3.66
*12:03	3	4	0.36	0.39	1	0.08	1.84
*14:02	4	2	1.20	1.00	1	0.20	6.00
*16:01	2	1	1.18	1.00	1	0.12	8.45
*16:02	1	0	1.85	0.53	1	0.10	15.01
*HLA-DQB1*	n=26	n=17					
*02:01	7	3	1.72	0.71	1	0.38	6.72
*02:02	3	3	0.61	0.67	1	0.12	3.12
*03:01	7	5	0.88	1.00	1	0.24	3.24
*03:02	2	2	0.63	1.00	1	0.10	4.08
*03:03	2	1	1.33	1.00	1	0.13	9.34
*04:02	5	2	1.79	0.68	1	0.31	8.11
*05:01	8	4	1.44	0.73	1	0.36	5.27
*05:02	1	1	0.64	1.00	1	0.06	6.76
*06:01	1	2	0.30	0.55	1	0.04	3.04
*06:02	6	3	1.40	1.00	1	0.30	5.67
*06:03	9	6	0.97	1.00	1	0.28	3.33
*06:04	1	2	0.30	0.55	1	0.04	3.04
*HLA-DRB1*	n=26	n=17					
*01:01	2	1	1.33	1.00	1	0.13	9.34
*01:02	4	2	1.36	1.00	1	0.23	6.63
*03:01	7	3	1.72	0.71	1	0.38	6.72
*04:01	1	0	2.06	0.51	1	0.12	16.44
*04:03	0	1	0.21	0.16	1	0.03	3.74
*04:04	2	1	1.33	1.00	1	0.13	9.34
*07:01	5	4	0.77	1.00	1	0.19	3.17
*08:01	5	2	1.79	0.68	1	0.31	8.11
*10:01	1	1	0.64	1.00	1	0.06	6.76
*11:01	5	4	0.77	1.00	1	0.19	3.17
*11:04	1	1	0.64	1.00	1	0.06	6.76
*13:01	9	6	0.97	1.00	1	0.28	3.33
*13:02	1	2	0.30	0.55	1	0.04	3.04
*15:01	7	3	1.72	0.71	1	0.38	6.72
*15:02	1	2	0.30	0.55	1	0.04	3.04
*16:01	1	1	0.64	1.00	1	0.06	6.76

**Figure 2:**
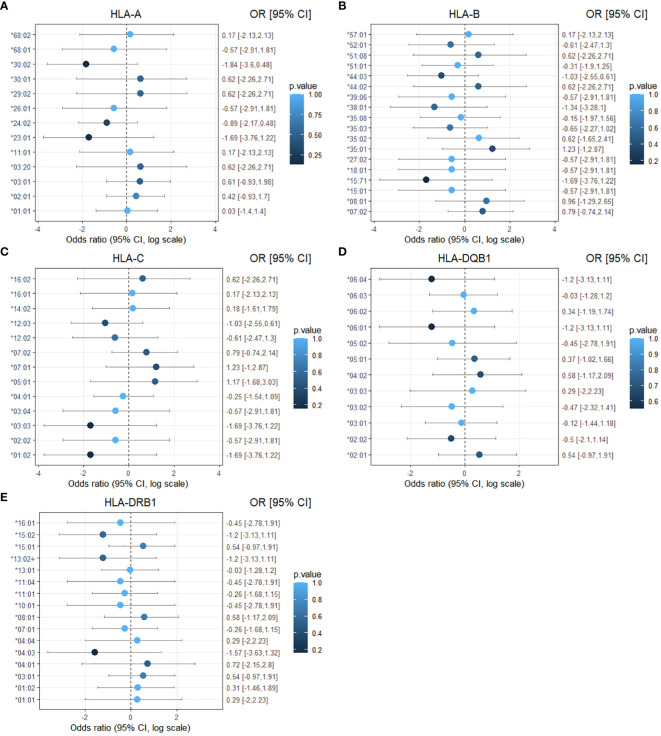
Forest plot of the association of HLA-I and HLA-II alleles with affected status of *CTLA4* mutation carriers. The results are displayed separately for five classes of HLA alleles: **(A)** HLA-A **(B)** HLA-B **(C)** HLA-C **(D)** HLA-DQB1 **(E)** HLA-DRB1. Dots represent the odds ratio and continuous lines represent the 95% confidence intervals in a logarithmic scale. Calculations were performed using the Haldane and Anscombe correction, and the adjusted Wald method for the confidence intervals. *p-values* are represented as color intensity.

**Figure 3:**
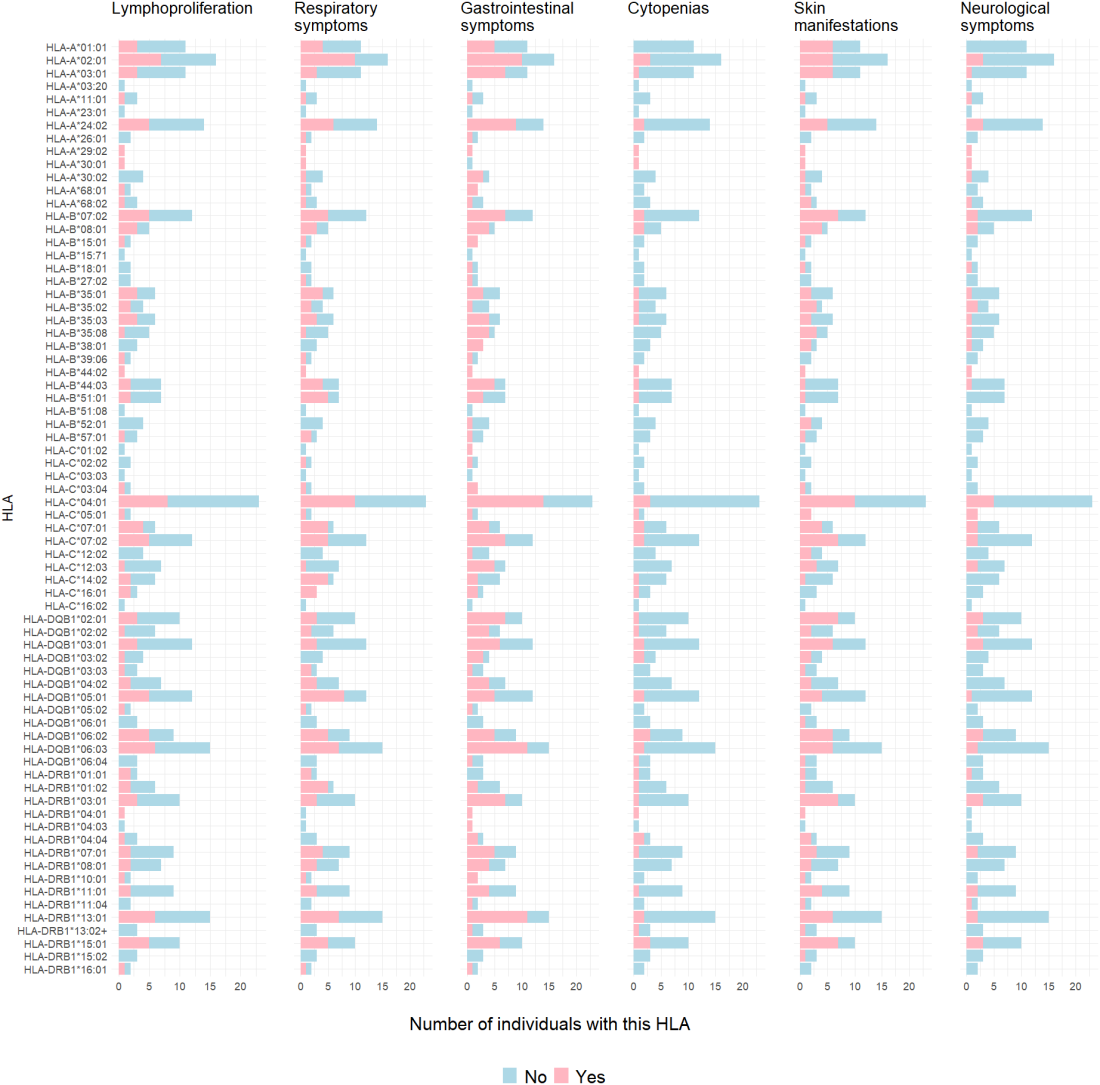
Clinical symptoms according to HLA class I or II in *CTLA4* mutation carriers. Bar graphs depict number of individuals with presence (pink) or absence (blue) of lymphoproliferation, cytopenias, respiratory, gastrointestinal, dermatological or neurological symptoms specific to HLA type.

No statistically significant difference in the odds of the outcome (affected status) between exposed and unexposed groups was observed for each HLA allele, with p-values > 0.05 (**Table 4**). To facilitate visualization and interpretation, OR and 95% CI were expressed on a logarithmic scale, which is symmetric around one (**Figure 2**). Positive values indicate positive associations (>0), and negative values indicate negative associations (<0). Similarly, OR analyses for disease severity showed no significant association with HLA alleles (**Supplementary Figure 1**, **Supplementary Table 1**). However, potential positive associations (raw p-values) of respiratory symptoms with HLA-DQB1*05:01 and HLA-DRB1*01:02 showed p-values <0.05 and 95% CI greater than one, indicating a trend that might be confirmed with increased sample size (**Supplementary Table 2**). HLA-DQB1*05:01 (OR 4.89, 95% CI: 1.14-17.63, p-value 0.04) was present in almost half of the individuals with respiratory involvement (8 out of 17 from five different families), while only 15% (4 out of 26 from four different families) individuals with no respiratory involvement carried this allele (**Figure 3**). HLA-DRB1*01:02 (OR 10.42, 95% CI: 1.09-51.43, p-value 0.03) was found in 29% (5 out of 17) of the subjects with respiratory manifestations but only in 4% (1 out of 26) of the subjects without respiratory symptoms (**Figure 3**). Noteworthy, all members carrying this HLA allele were from family D. A third HLA-allele, HLA-C*05:01, showed a positive association with neurological symptoms with a raw p-value of 0.04 and 28.64 odds ratio (95% CI: 1.42-180.9). Only seven patients exhibited neurological symptoms, from whom two, belonging to different families, carried this allele (**Supplementary Table 3** and **Figure 3**). Finally, the HLA-DRB1*01:01 allele demonstrated a protective association with gastrointestinal symptoms (OR 0.1, 95% CI: 0.02-1.66, p-value 0.04, **Supplementary Table 4**). This allele was found in two affected individuals from two separate families, and one unaffected individual from one of these families. Importantly, none of the 24 individuals who experienced gastrointestinal symptoms carried this allele (**Figure 3**). While other associations were identified with p-values < 0.05, the confidence intervals overlapped the null value, and the alleles were present in only one individual or a single family. These associations included HLA-C*16:01 with respiratory tract involvement; HLA-A*29:02, HLA-A*30:01, and HLA-B*44:02 with neurological manifestations; HLA-DQB1*06:01 and HLA-DRB1*15:02 with gastrointestinal symptoms; HLA-A*29:02, HLA-A*30:01, HLA-B*44:02 and HLA-DRB1*04:01 with cytopenias (**Supplementary Tables 2**-**6**).

### 3.5 Genetic linkage analysis of the HLA locus

We did genetic linkage analysis to assess the possibility that there is linkage to the HLA locus in the absence of allele-specific association. Linkage without association can occur if different (HLA) alleles are co-inherited with the diseases in different families. The power to detect different LOD scores was calculated for four different settings of the frequency of the disease-associated allele at the modifier locus, using a single 5-allele marker locus (**Table 5**). While the power to detect a statistically significant LOD score > 3.0 is low for single markers, the power to detect a positive score > 1.0 is high and this can be boosted by using multi-marker analysis.

**Table 5:** Power to detect LOD scores.

LOD score thresh/allele freq.	0.01, no phenocopy	0.10, phenocopy	0.20 phenocopy	0.50 phenocopy
1.0	0.91	0.72	0.66	0.45
2.0	0.74	0.47	0.36	0.16
3.0	0.57	0.30	0.18	0.06

Power to detect a LOD score greater than 1.0, 2.0., 3.0 for four different settings of the frequency of the disease-associated allele at the modifier locus, using a single 5-allele marker locus. The maximum possible value would be 1.00. Each column is based on 100 replicates.

All LOD scores were negative, indicating the absence of genetic linkage (**Table 6**). Therefore, for simplicity, we show the scores at a recombination fraction of 0 between the putative modifier locus and the marker locus. It is not surprising that the observed LOD scores for four out of five marker loci are almost identical because the marker loci are linked and hence have equivalent patterns of inheritance of the marker alleles. The HLA-C locus is less informative than the other four loci and hence the negative scores at HLA-C have a lower magnitude, but also indicate the absence of linkage.

**Table 6:** Observed scores.

LOD score/allele freq.	0.01, no phenocopy	0.10, phenocopy	0.20, phenocopy	0.50, phenocopy
HLA-A	-11.39	-5.38	-4.20	-2.37
HLA-B	-11.40	-5.30	-4.16	-2.33
HLA-C	-6.07	-3.37	-2.81	-1.77
HLA-DRB1	-11.38	-5.42	-4.28	-2.41
HLA-DRQ1	-11.37	-5.34	-4.17	-2.36

Observed LOD scores for each of the five HLA loci that were genotyped and for four choices of the possible frequency of the disease-associated marker allele.

## 4 Discussion

In this exploratory study, we evaluated the frequency of HLA class I and class II alleles as a potential genetic disease modifier factor in CTLA-4 insufficiency. In particular, we analyzed the frequency of HLA alleles A, B, C, DRB1, and DQB1 in 43 *CTLA4* mutation carriers coming from seven different families. Our findings indicated no statistically significant associations between the frequency of HLA alleles and the disease onset, or the disease severity observed in individuals carrying genetic variants in *CTLA4*. However, we found potential positive associations of HLA-DQB1*05:01 and HLA-DRB1*01:02 alleles with respiratory tract involvement. It is noteworthy that all individuals with the HLA-DRB1*01:02 allele were from the same family, suggesting that other genetic or environmental factors may be influencing these findings. Importantly, HLA-DRB1*01:02 has been associated with susceptibility to rheumatoid arthritis (27), and HLA-DQB1*05:01 with a cluster of severe Guillain-Barré syndrome (28). Additionally, a potential positive association of HLA-C*05:01 with neurological involvement was observed, an allele associated with COVID-19 mortality (29). Interestingly, HLA-DRB1*01:01 showed a protective association with gastrointestinal symptoms. This allele has been reported to be protective for multiple sclerosis (30) and for the development of antibodies against administered factor VIII therapy in hemophilia A (31). Further investigation of HLA type I and II alleles in a larger sample size is warranted to confirm any significant correlation.

It is important to acknowledge various critical limitations in our study. First, the small number of CTLA-4 insufficient families analyzed resulted in a low statistical power in both, the single HLA allele frequencies association and the genetic linkage analysis methods, indicating that there is only a small chance of detecting a true effect of the HLA alleles. Furthermore, the odds ratio calculations did not account for the familial clustering of some individuals, which could have influenced the results. Second, the small number of HLA alleles analyzed in our patient cohort. HLA genes have a high number of polymorphisms that can be better detected using a genome wide association study (GWAS) instead of four-digit HLA typing. Finally, the lack of a long-term follow-up of the *CTLA4* mutations carriers is a limitation, as they might change their disease status over time. However, it is worth noting that these constraints are common in studies involving rare diseases such as CTLA-4 insufficiency, where, to our knowledge, only 222 patients have been published to date (32) making these challenges exceedingly difficult to overcome.

To date, several additional modifiers in the context of CTLA-4 insufficiency have been suggested, but none has shown a positive association. For instance, infections with specific pathogens reported no difference between *affected* and *unaffected CTLA4* mutation carriers, specifically for the seroprevalence for EBV (EBNA-1) IgG, CMV IgG, parvovirus B19 IgG, HSV-1/2 IgG and *Toxoplasma gondii*. Similar negative associations were found with EBV-specific IgG antibodies measured by FcgRIII/CD16A activation (8). Interestingly, HLA class II plays an important role in the entry of EBV into B cells. In fact, HLA-DQ isotypes show variable binding affinity for the EBV envelope glycoprotein gp42, supporting the association between susceptibility and resistance to EBV according to the HLA-DQ isotype (33). As previous reports revealed a particular susceptibility of CTLA-4 insufficient patients to EBV infections (34), we cannot exclude the hypothesis that interaction between a specific HLA type and an infectious pathogen trigger the disease onset or disease severity in CTLA-4 insufficiency.

Fifty years since the first report on the association between HLA-B and Hodgkin’s lymphoma, HLA alleles have been associated with a great number of human diseases, proving that HLA molecules are essential for protective immunity and for deleterious, disease-causing autoimmune reactivity (9). The current molecular understanding behind this association lays on two major mechanisms. The first one is the specificity of HLA-peptide-TCR interaction, which is governed by several mechanisms such as alternate TCR docking, low-affinity-mediated thymic escape, TCR stabilization of a weak peptide-HLA complexes, and altered presentation for peptides. The second major mechanism that might also generate and activate autoreactive T cells is epitope variation that includes, molecular mimicry, post-translational modification of antigenic peptides, generation of hybrid peptides, and differential HLA expression and stability (35). Both mechanisms generate autoreactive T cells that escape negative selection in the thymus and in the periphery, infiltrating organs and causing tissue damage. However, it remains to be determined whether these autoreactive T cells are a consequence of altered multiple mechanisms in a single patient, or whether certain molecular mechanisms are shared in a particular diseases, or in patient subgroups carrying the same HLA risk alleles. Moreover, it remains challenging to identify a particular self-peptide that becomes immunogenic during lifetime and causes activation of autoreactive T cells in a specific autoimmune response. Several technical approaches addressing this challenge have been developed, including the identification of clonally expanded and presumably pathogenic CD4^+^ or CD8^+^ T cells by single cell TCR analysis in the organ-infiltrated tissue, or characterization of the peptide recognition motif of the TCRs of interest by peptide library, or screening of the immunopeptidome. Analysis of the TCR repertoire of tissue-infiltrating T cells in CTLA-4 insufficient patients will be beneficial.

In addition to genetic disease triggers, several researchers have proposed the potential effect of non-genetic modifiers such as epigenetic changes or environmental factors like observed in other IEI. For instance, hypermethylation of various B-cell genes were found as a disease factor in a pair of monozygotic twins discordant for CVID (36). Moreover, *Ramirez et al.*, identified different chromatin landscapes involving binding sites for transcription factors (TF) known for their importance in the germinal center reaction, in patients with diagnosis of CVID and mutations in *TNFRSF13B* from healthy donors (37). Gut dysbiosis is another possible disease modifier in CTLA-4 insufficiency. Several studies have shown a positive association between gut dysbiosis and systemic inflammation in several IEI, including chronic granulomatous disease (CGD), selective IgA deficiency, X-linked lymphoproliferative disease type-2 (XLP) and in CVID (38–40). It is thought that dysfunction of gut barriers due to damaged cell tight junctions, result in increased exposure of HLA to specific pathogens in the gut (41).

In conclusion, our results suggest that HLA-A, -B, -C, DRB1, DQB1 are unlikely to trigger the disease onset in CTLA-4 insufficient individuals and are not correlated with disease severity. However, our investigation raises the possibility that HLA-DQB1*05:01, HLA-DRB1*01:02, HLA-C*05:01 and HLA-DRB1*01:01 may influence the development of certain symptoms. It is important to note that this study is exploratory, and future studies with larger cohorts may either refute these findings or uncover other associations with specific clinical presentations. Additionally, further research, including epigenetic and microbiome analyses in a larger cohort of *CTLA4* mutation carriers, will contribute to understand the pathogenicity of CTLA-4 insufficiency.

## Data availability statement

The datasets presented in this article are not readily available due to ethical and privacy restrictions. Requests to access the datasets should be directed to bodo.grimbacher@uniklinik-freiburg.de.

## Ethics statement

The studies involving humans were approved by Ethics Committee, Medical Centre - University of Freiburg, (registration number 466/18). The studies were conducted in accordance with the local legislation and institutional requirements. Written informed consent for participation in this study was provided by the participants or the participants’ legal guardians/next of kin.

## Author contributions

SP-C: Data curation, Formal analysis, Investigation, Methodology, Software, Visualization, Writing – original draft, Writing – review & editing. NM: Conceptualization, Data curation, Investigation, Methodology, Project administration, Writing – original draft, Writing – review & editing. FE: Investigation, Methodology, Writing – original draft, Writing – review & editing. VP: Data curation, Resources, Writing – original draft, Writing – review & editing. H-ML: Data curation, Resources, Writing – original draft, Writing – review & editing. ON: Data curation, Resources, Writing – original draft, Writing – review & editing. ID: Data curation, Resources, Writing – original draft, Writing – review & editing. KT: Data curation, Resources, Writing – original draft, Writing – review & editing. AAS: Formal analysis, Investigation, Methodology, Software, Writing – original draft, Writing – review & editing. BG: Conceptualization, Funding acquisition, Project administration, Resources, Supervision, Writing – original draft, Writing – review & editing. LG-D: Conceptualization, Data curation, Formal analysis, Funding acquisition, Investigation, Methodology, Project administration, Software, Supervision, Visualization, Writing – original draft, Writing – review & editing.
