# Supplementary material for: The effect of HLA genotype on disease onset and severity in CTLA-4 insufficiency
Source: Front Immunol. 2025 Jan 6;15:1447995. doi: 10.3389/fimmu.2024.1447995 (PMC11744039; doi:10.3389/fimmu.2024.1447995)
Supplement: Supplementary file 1 [file Table1.docx]

# Supplementary Figures

**
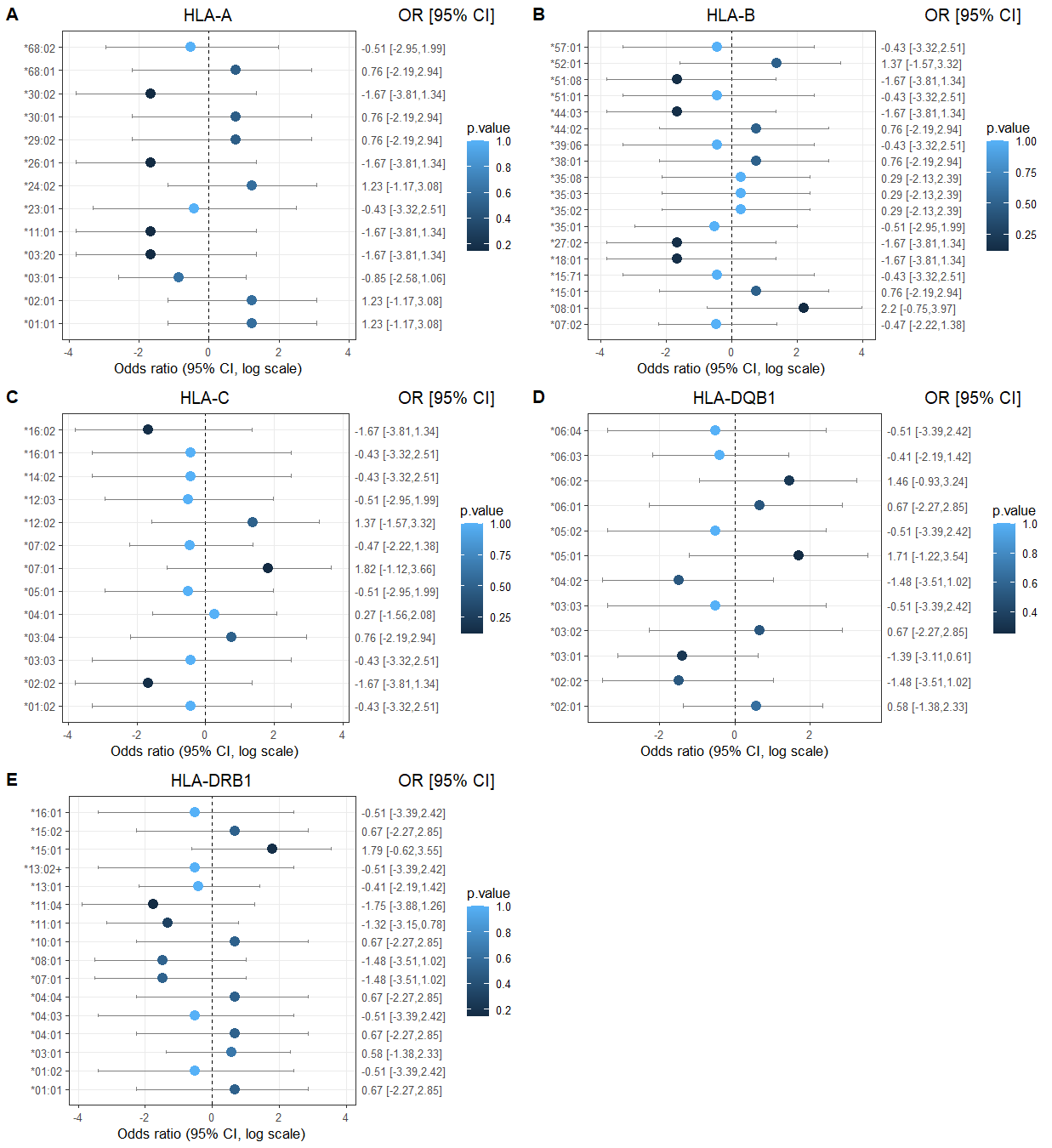
**

**Supplementary Figure 1. Forest plot of the association of HLA-I and HLA-II alleles with disease severity in affected *CTLA4* mutation carriers**. Dots represent the odds ratio and continuous lines represent the 95% confidence intervals in a logarithmic scale. Calculations were performed using the Haldane and Anscombe correction and the adjusted Wald test for the confidence intervals. *p-values* are represented as color intensity.

# Supplementary Tables

## Supplementary Table 1. Association of HLA-I and HLA-II alleles with disease severity in affected *CTLA4* mutation carriers

| **Allele** | **Severely affected** | **Mildly affected** | **Odds.Ratio** | **p.value** | **p.adjusted**  **(Bonferroni)** | **Lower.CI** | **Upper.CI** |
| --- | --- | --- | --- | --- | --- | --- | --- |
| **HLA-A** | n=11 | n=7 |  |  |  |  |  |
| *01:01 | 4 | 1 | 3,43 | 0,60 | 1 | 0,31 | 21,78 |
| *02:01 | 4 | 1 | 3,43 | 0,60 | 1 | 0,31 | 21,78 |
| *03:01 | 4 | 4 | 0,43 | 0,63 | 1 | 0,08 | 2,88 |
| *03:20 | 0 | 1 | 0,19 | 0,15 | 1 | 0,02 | 3,83 |
| *11:01 | 0 | 1 | 0,19 | 0,15 | 1 | 0,02 | 3,83 |
| *23:01 | 0 | 0 | 0,65 | 1,00 | 1 | 0,04 | 12,27 |
| *24:02 | 4 | 1 | 3,43 | 0,60 | 1 | 0,31 | 21,78 |
| *26:01 | 0 | 1 | 0,19 | 0,15 | 1 | 0,02 | 3,83 |
| *29:02 | 1 | 0 | 2,14 | 0,49 | 1 | 0,11 | 18,96 |
| *30:01 | 1 | 0 | 2,14 | 0,49 | 1 | 0,11 | 18,96 |
| *30:02 | 0 | 1 | 0,19 | 0,15 | 1 | 0,02 | 3,83 |
| *68:01 | 1 | 0 | 2,14 | 0,49 | 1 | 0,11 | 18,96 |
| *68:02 | 1 | 1 | 0,60 | 1,00 | 1 | 0,05 | 7,29 |
| **HLA-B** | n=11 | n=7 |  |  |  |  |  |
| *07:02 | 5 | 4 | 0,63 | 1,00 | 1 | 0,11 | 3,98 |
| *08:01 | 4 | 0 | 9 | 0,12 | 1 | 0,47 | 52,96 |
| *15:01 | 1 | 0 | 2,14 | 0,49 | 1 | 0,11 | 18,96 |
| *15:71 | 0 | 0 | 0,65 | 1,00 | 1 | 0,04 | 12,27 |
| *18:01 | 0 | 1 | 0,19 | 0,15 | 1 | 0,02 | 3,83 |
| *27:02 | 0 | 1 | 0,19 | 0,15 | 1 | 0,02 | 3,83 |
| *35:01 | 1 | 1 | 0,60 | 1,00 | 1 | 0,05 | 7,29 |
| *35:02 | 2 | 1 | 1,33 | 1,00 | 1 | 0,12 | 10,89 |
| *35:03 | 2 | 1 | 1,33 | 1,00 | 1 | 0,12 | 10,89 |
| *35:08 | 2 | 1 | 1,33 | 1,00 | 1 | 0,12 | 10,89 |
| *38:01 | 1 | 0 | 2,14 | 0,49 | 1 | 0,11 | 18,96 |
| *39:06 | 0 | 0 | 0,65 | 1,00 | 1 | 0,04 | 12,27 |
| *44:02 | 1 | 0 | 2,14 | 0,49 | 1 | 0,11 | 18,96 |
| *44:03 | 0 | 1 | 0,19 | 0,15 | 1 | 0,02 | 3,83 |
| *51:01 | 0 | 0 | 0,65 | 1,00 | 1 | 0,04 | 12,27 |
| *51:08 | 0 | 1 | 0,19 | 0,15 | 1 | 0,02 | 3,83 |
| *52:01 | 2 | 0 | 3,95 | 0,49 | 1 | 0,21 | 27,72 |
| *57:01 | 0 | 0 | 0,65 | 1,00 | 1 | 0,04 | 12,27 |
| **HLA-C** | n=11 | n=7 |  |  |  |  |  |
| *01:02 | 0 | 0 | 0,65 | 1,00 | 1 | 0,04 | 12,27 |
| *02:02 | 0 | 1 | 0,19 | 0,15 | 1 | 0,02 | 3,83 |
| *03:03 | 0 | 0 | 0,65 | 1,00 | 1 | 0,04 | 12,27 |
| *03:04 | 1 | 0 | 2,14 | 0,49 | 1 | 0,11 | 18,96 |
| *04:01 | 7 | 4 | 1,31 | 1,00 | 1 | 0,21 | 8,01 |
| *05:01 | 1 | 1 | 0,60 | 1,00 | 1 | 0,05 | 7,29 |
| *07:01 | 3 | 0 | 6,18 | 0,12 | 1 | 0,33 | 38,78 |
| *07:02 | 5 | 4 | 0,63 | 1,00 | 1 | 0,11 | 3,98 |
| *12:02 | 2 | 0 | 3,95 | 0,49 | 1 | 0,21 | 27,72 |
| *12:03 | 1 | 1 | 0,60 | 1,00 | 1 | 0,05 | 7,29 |
| *14:02 | 0 | 0 | 0,65 | 1,00 | 1 | 0,04 | 12,27 |
| *16:01 | 0 | 0 | 0,65 | 1,00 | 1 | 0,04 | 12,27 |
| *16:02 | 0 | 1 | 0,19 | 0,15 | 1 | 0,02 | 3,83 |
| **HLA-DQB1** | n=12 | n=7 |  |  |  |  |  |
| *02:01 | 5 | 2 | 1,79 | 0,66 | 1 | 0,25 | 10,32 |
| *02:02 | 1 | 2 | 0,23 | 0,52 | 1 | 0,03 | 2,76 |
| *03:01 | 3 | 4 | 0,25 | 0,33 | 1 | 0,04 | 1,84 |
| *03:02 | 1 | 0 | 1,96 | 0,52 | 1 | 0,10 | 17,28 |
| *03:03 | 0 | 0 | 0,60 | 1,00 | 1 | 0,03 | 11,28 |
| *04:02 | 1 | 2 | 0,23 | 0,52 | 1 | 0,03 | 2,76 |
| *05:01 | 3 | 0 | 5,53 | 0,25 | 1 | 0,30 | 34,59 |
| *05:02 | 0 | 0 | 0,60 | 1,00 | 1 | 0,03 | 11,28 |
| *06:01 | 1 | 0 | 1,96 | 0,52 | 1 | 0,10 | 17,28 |
| *06:02 | 5 | 1 | 4,29 | 0,33 | 1 | 0,39 | 25,66 |
| *06:03 | 4 | 3 | 0,67 | 1,00 | 1 | 0,11 | 4,14 |
| *06:04 | 0 | 0 | 0,60 | 1,00 | 1 | 0,03 | 11,28 |
| **HLA-DRB1** | n=11 | n=7 |  |  |  |  |  |
| *01:01 | 1 | 0 | 1,96 | 0,52 | 1 | 0,10 | 17,28 |
| *01:02 | 0 | 0 | 0,60 | 1,00 | 1 | 0,03 | 11,28 |
| *03:01 | 5 | 2 | 1,79 | 0,66 | 1 | 0,25 | 10,32 |
| *04:01 | 1 | 0 | 1,96 | 0,52 | 1 | 0,10 | 17,28 |
| *04:03 | 0 | 0 | 0,60 | 1,00 | 1 | 0,03 | 11,28 |
| *04:04 | 1 | 0 | 1,96 | 0,52 | 1 | 0,10 | 17,28 |
| *07:01 | 1 | 2 | 0,23 | 0,52 | 1 | 0,03 | 2,76 |
| *08:01 | 1 | 2 | 0,23 | 0,52 | 1 | 0,03 | 2,76 |
| *10:01 | 1 | 0 | 1,96 | 0,52 | 1 | 0,10 | 17,28 |
| *11:01 | 2 | 3 | 0,27 | 0,30 | 1 | 0,04 | 2,18 |
| *11:04 | 0 | 1 | 0,17 | 0,15 | 1 | 0,02 | 3,52 |
| *13:01 | 4 | 3 | 0,67 | 1,00 | 1 | 0,11 | 4,14 |
| *13:02 | 0 | 0 | 0,60 | 1,00 | 1 | 0,03 | 11,28 |
| *15:01 | 6 | 1 | 6 | 0,17 | 1 | 0,54 | 34,75 |
| *15:02 | 1 | 0 | 1,96 | 0,52 | 1 | 0,10 | 17,28 |
| *16:01 | 0 | 0 | 0,60 | 1,00 | 1 | 0,03 | 11,28 |

## Supplementary Table 2. Association of HLA-I and HLA-II alleles with respiratory tract involvement in *CTLA4* mutation carriers

| **Allele** | **Respiratory tract involvement** | **No respiratory tract involvement** | **Odds.Ratio** | **p.value** | **p.adjusted**  **(Bonferroni)** | **Lower.CI** | **Upper.CI** |
| --- | --- | --- | --- | --- | --- | --- | --- |
| ***HLA-A*** | n=17 | n=23 |  |  |  |  |  |
| *01:01 | 4 | 7 | 0,62 | 0,72 | 1,00 | 0,16 | 2,57 |
| *02:01 | 10 | 6 | 3,57 | 0,10 | 1,00 | 0,90 | 12,38 |
| *03:01 | 3 | 8 | 0,35 | 0,28 | 1,00 | 0,09 | 1,63 |
| *03:20 | 0 | 1 | 0,39 | 0,49 | 1,00 | 0,05 | 6,98 |
| *11:01 | 1 | 2 | 0,59 | 1,00 | 1,00 | 0,08 | 5,95 |
| *23:01 | 0 | 1 | 0,39 | 0,49 | 1,00 | 0,05 | 6,98 |
| *24:02 | 6 | 8 | 0,89 | 1,00 | 1,00 | 0,25 | 3,26 |
| *26:01 | 1 | 1 | 1,25 | 1,00 | 1,00 | 0,12 | 13,08 |
| *29:02 | 1 | 0 | 3,91 | 0,20 | 1,00 | 0,22 | 30,98 |
| *30:01 | 1 | 0 | 3,91 | 0,20 | 1,00 | 0,22 | 30,98 |
| *30:02 | 1 | 3 | 0,38 | 0,61 | 1,00 | 0,06 | 3,64 |
| *68:01 | 1 | 1 | 1,25 | 1,00 | 1,00 | 0,12 | 13,08 |
| *68:02 | 1 | 2 | 0,59 | 1,00 | 1,00 | 0,08 | 5,95 |
| ***HLA-B*** | n=17 | n=23 |  |  |  |  |  |
| *07:02 | 5 | 7 | 0,83 | 1,00 | 1,00 | 0,22 | 3,23 |
| *08:01 | 3 | 2 | 2,04 | 0,64 | 1,00 | 0,32 | 10,94 |
| *15:01 | 1 | 1 | 1,25 | 1,00 | 1,00 | 0,12 | 13,08 |
| *15:71 | 0 | 1 | 0,39 | 0,49 | 1,00 | 0,05 | 6,98 |
| *18:01 | 0 | 2 | 0,22 | 0,49 | 1,00 | 0,04 | 3,89 |
| *27:02 | 1 | 1 | 1,25 | 1,00 | 1,00 | 0,12 | 13,08 |
| *35:01 | 4 | 2 | 2,92 | 0,38 | 1,00 | 0,48 | 14,16 |
| *35:02 | 2 | 2 | 1,27 | 1,00 | 1,00 | 0,19 | 8,21 |
| *35:03 | 3 | 3 | 1,29 | 1,00 | 1,00 | 0,25 | 6,53 |
| *35:08 | 1 | 4 | 0,27 | 0,36 | 1,00 | 0,05 | 2,53 |
| *38:01 | 0 | 3 | 0,15 | 0,11 | 1,00 | 0,03 | 2,59 |
| *39:06 | 1 | 1 | 1,25 | 1,00 | 1,00 | 0,12 | 13,08 |
| *44:02 | 1 | 0 | 3,91 | 0,20 | 1,00 | 0,22 | 30,98 |
| *44:03 | 4 | 3 | 1,85 | 0,68 | 1,00 | 0,37 | 8,41 |
| *51:01 | 5 | 2 | 3,96 | 0,21 | 1,00 | 0,66 | 17,96 |
| *51:08 | 0 | 1 | 0,39 | 0,49 | 1,00 | 0,05 | 6,98 |
| *52:01 | 0 | 4 | 0,11 | 0,11 | 1,00 | 0,02 | 1,89 |
| *57:01 | 2 | 1 | 2,67 | 0,58 | 1,00 | 0,26 | 18,53 |
| ***HLA-C*** | n=17 | n=23 |  |  |  |  |  |
| *01:02 | 0 | 1 | 0,39 | 0,49 | 1,00 | 0,05 | 6,98 |
| *02:02 | 1 | 1 | 1,25 | 1,00 | 1,00 | 0,12 | 13,08 |
| *03:03 | 0 | 1 | 0,39 | 0,49 | 1,00 | 0,05 | 6,98 |
| *03:04 | 1 | 1 | 1,25 | 1,00 | 1,00 | 0,12 | 13,08 |
| *04:01 | 10 | 13 | 0,88 | 1,00 | 1,00 | 0,25 | 3,14 |
| *05:01 | 1 | 1 | 1,25 | 1,00 | 1,00 | 0,12 | 13,08 |
| *07:01 | 5 | 1 | 8,33 | 0,07 | 0,93 | 0,87 | 41,74 |
| *07:02 | 5 | 7 | 0,83 | 1,00 | 1,00 | 0,22 | 3,23 |
| *12:02 | 0 | 4 | 0,11 | 0,11 | 1,00 | 0,02 | 1,89 |
| *12:03 | 1 | 6 | 0,16 | 0,10 | 1,00 | 0,03 | 1,46 |
| *14:02 | 5 | 1 | 8,33 | 0,07 | 0,93 | 0,87 | 41,74 |
| *16:01 | 3 | 0 | 10,38 | 0,03 | 0,44 | 0,60 | 57,79 |
| *16:02 | 0 | 1 | 0,39 | 0,49 | 1,00 | 0,05 | 6,98 |
| ***HLA-DQB1*** | n=17 | n=26 |  |  |  |  |  |
| *02:01 | 3 | 7 | 0,58 | 0,71 | 1,00 | 0,15 | 2,65 |
| *02:02 | 2 | 4 | 0,73 | 1,00 | 1,00 | 0,15 | 4,31 |
| *03:01 | 3 | 9 | 0,40 | 0,31 | 1,00 | 0,11 | 1,82 |
| *03:02 | 0 | 4 | 0,14 | 0,13 | 1,00 | 0,03 | 2,39 |
| *03:03 | 2 | 1 | 3,33 | 0,55 | 1,00 | 0,33 | 22,85 |
| *04:02 | 3 | 4 | 1,18 | 1,00 | 1,00 | 0,26 | 5,66 |
| *05:01 | 8 | 4 | 4,89 | 0,04 | 0,45 | 1,14 | 17,63 |
| *05:02 | 1 | 1 | 1,56 | 1,00 | 1,00 | 0,15 | 16,15 |
| *06:01 | 0 | 3 | 0,19 | 0,14 | 1,00 | 0,03 | 3,24 |
| *06:02 | 5 | 4 | 2,29 | 0,44 | 1,00 | 0,53 | 9,15 |
| *06:03 | 7 | 8 | 1,58 | 0,53 | 1,00 | 0,45 | 5,37 |
| *06:04 | 0 | 3 | 0,19 | 0,14 | 1,00 | 0,03 | 3,24 |
| ***HLA-DRB1*** | n=17 | n=26 |  |  |  |  |  |
| *01:01 | 2 | 1 | 3,33 | 0,55 | 1,00 | 0,33 | 22,85 |
| *01:02 | 5 | 1 | 10,42 | 0,03 | 0,45 | 1,09 | 51,43 |
| *03:01 | 3 | 7 | 0,58 | 0,71 | 1,00 | 0,15 | 2,65 |
| *04:01 | 0 | 1 | 0,49 | 0,51 | 1,00 | 0,06 | 8,58 |
| *04:03 | 0 | 1 | 0,49 | 0,51 | 1,00 | 0,06 | 8,58 |
| *04:04 | 0 | 3 | 0,19 | 0,14 | 1,00 | 0,03 | 3,24 |
| *07:01 | 4 | 5 | 1,29 | 1,00 | 1,00 | 0,32 | 5,39 |
| *08:01 | 3 | 4 | 1,18 | 1,00 | 1,00 | 0,26 | 5,66 |
| *10:01 | 1 | 1 | 1,56 | 1,00 | 1,00 | 0,15 | 16,15 |
| *11:01 | 3 | 6 | 0,71 | 1,00 | 1,00 | 0,18 | 3,29 |
| *11:04 | 0 | 2 | 0,28 | 0,51 | 1,00 | 0,04 | 4,82 |
| *13:01 | 7 | 8 | 1,58 | 0,53 | 1,00 | 0,45 | 5,37 |
| *13:02 | 0 | 3 | 0,19 | 0,14 | 1,00 | 0,03 | 3,24 |
| *15:01 | 5 | 5 | 1,75 | 0,48 | 1,00 | 0,44 | 6,78 |
| *15:02 | 0 | 3 | 0,19 | 0,14 | 1,00 | 0,03 | 3,24 |
| *16:01 | 1 | 1 | 1,56 | 1,00 | 1,00 | 0,15 | 16,15 |

## Supplementary Table 3. Association of HLA-I and HLA-II alleles with neurological involvement in *CTLA4* mutation carriers

| **Allele** | **Neurological involvement** | **No neurological involvement** | **Odds.Ratio** | **p.value** | **p.adjusted**  **(Bonferroni)** | **Lower.CI** | **Upper.CI** |
| --- | --- | --- | --- | --- | --- | --- | --- |
| ***HLA-A*** | n=7 | n=31 |  |  |  |  |  |
| *01:01 | 0 | 11 | 0,12 | 0,08 | 1,00 | 0,02 | 1,97 |
| *02:01 | 3 | 13 | 1,04 | 1,00 | 1,00 | 0,22 | 5,09 |
| *03:01 | 1 | 10 | 0,35 | 0,65 | 1,00 | 0,07 | 3,23 |
| *03:20 | 0 | 1 | 1,36 | 1,00 | 1,00 | 0,16 | 24,16 |
| *11:01 | 1 | 2 | 2,42 | 0,47 | 1,00 | 0,30 | 24,46 |
| *23:01 | 0 | 1 | 1,36 | 1,00 | 1,00 | 0,16 | 24,16 |
| *24:02 | 3 | 11 | 1,36 | 1,00 | 1,00 | 0,29 | 6,68 |
| *26:01 | 0 | 2 | 0,79 | 1,00 | 1,00 | 0,11 | 13,69 |
| *29:02 | 1 | 0 | 14,54 | 0,04 | 0,47 | 0,72 | 115,46 |
| *30:01 | 1 | 0 | 14,54 | 0,04 | 0,47 | 0,72 | 115,46 |
| *30:02 | 1 | 3 | 1,56 | 1,00 | 1,00 | 0,23 | 15,25 |
| *68:01 | 0 | 2 | 0,79 | 1,00 | 1,00 | 0,11 | 13,69 |
| *68:02 | 1 | 2 | 2,42 | 0,47 | 1,00 | 0,30 | 24,46 |
| ***HLA-B*** | n=7 | n=31 |  |  |  |  |  |
| *07:02 | 2 | 10 | 0,84 | 1,00 | 1,00 | 0,18 | 4,93 |
| *08:01 | 2 | 3 | 3,73 | 0,22 | 1,00 | 0,57 | 23,83 |
| *15:01 | 0 | 2 | 0,79 | 1,00 | 1,00 | 0,11 | 13,69 |
| *15:71 | 0 | 1 | 1,36 | 1,00 | 1,00 | 0,16 | 24,16 |
| *18:01 | 1 | 1 | 5 | 0,34 | 1,00 | 0,42 | 52,58 |
| *27:02 | 0 | 2 | 0,79 | 1,00 | 1,00 | 0,11 | 13,69 |
| *35:01 | 1 | 5 | 0,87 | 1,00 | 1,00 | 0,15 | 8,21 |
| *35:02 | 2 | 2 | 5,80 | 0,15 | 1,00 | 0,74 | 38,67 |
| *35:03 | 1 | 5 | 0,87 | 1,00 | 1,00 | 0,15 | 8,21 |
| *35:08 | 1 | 4 | 1,13 | 1,00 | 1,00 | 0,18 | 10,80 |
| *38:01 | 1 | 2 | 2,42 | 0,47 | 1,00 | 0,30 | 24,46 |
| *39:06 | 0 | 2 | 0,79 | 1,00 | 1,00 | 0,11 | 13,69 |
| *44:02 | 1 | 0 | 14,54 | 0,04 | 0,65 | 0,72 | 115,46 |
| *44:03 | 1 | 6 | 0,69 | 1,00 | 1,00 | 0,13 | 6,51 |
| *51:01 | 0 | 7 | 0,22 | 0,17 | 1,00 | 0,04 | 3,62 |
| *51:08 | 0 | 1 | 1,36 | 1,00 | 1,00 | 0,16 | 24,16 |
| *52:01 | 0 | 4 | 0,41 | 0,57 | 1,00 | 0,07 | 6,89 |
| *57:01 | 0 | 3 | 0,54 | 0,57 | 1,00 | 0,09 | 9,29 |
| ***HLA-C*** | n=7 | n=31 |  |  |  |  |  |
| *01:02 | 0 | 1 | 1,36 | 1,00 | 1,00 | 0,16 | 24,16 |
| *02:02 | 0 | 2 | 0,79 | 1,00 | 1,00 | 0,11 | 13,69 |
| *03:03 | 0 | 1 | 1,36 | 1,00 | 1,00 | 0,16 | 24,16 |
| *03:04 | 0 | 2 | 0,79 | 1,00 | 1,00 | 0,11 | 13,69 |
| *04:01 | 5 | 18 | 1,81 | 0,68 | 1,00 | 0,31 | 8,37 |
| *05:01 | 2 | 0 | 28,64 | 0,04 | 0,47 | 1,42 | 180,90 |
| *07:01 | 2 | 4 | 2,70 | 0,30 | 1,00 | 0,46 | 16,75 |
| *07:02 | 2 | 10 | 0,84 | 1,00 | 1,00 | 0,18 | 4,93 |
| *12:02 | 0 | 4 | 0,41 | 0,57 | 1,00 | 0,07 | 6,89 |
| *12:03 | 2 | 5 | 2,08 | 0,59 | 1,00 | 0,38 | 12,66 |
| *14:02 | 0 | 6 | 0,26 | 0,32 | 1,00 | 0,05 | 4,36 |
| *16:01 | 0 | 3 | 0,54 | 0,57 | 1,00 | 0,09 | 9,29 |
| *16:02 | 0 | 1 | 1,36 | 1,00 | 1,00 | 0,16 | 24,16 |
| ***HLA-DQB1*** | n=7 | n=36 |  |  |  |  |  |
| *02:01 | 3 | 7 | 3,11 | 0,32 | 1,00 | 0,61 | 15,31 |
| *02:02 | 2 | 4 | 3,20 | 0,25 | 1,00 | 0,55 | 19,68 |
| *03:01 | 3 | 9 | 2,25 | 0,38 | 1,00 | 0,46 | 10,93 |
| *03:02 | 0 | 4 | 0,48 | 1,00 | 1,00 | 0,08 | 8,08 |
| *03:03 | 0 | 3 | 0,64 | 1,00 | 1,00 | 0,10 | 10,84 |
| *04:02 | 0 | 7 | 0,26 | 0,32 | 1,00 | 0,05 | 4,32 |
| *05:01 | 1 | 11 | 0,38 | 0,65 | 1,00 | 0,08 | 3,45 |
| *05:02 | 0 | 2 | 0,92 | 1,00 | 1,00 | 0,13 | 15,91 |
| *06:01 | 0 | 3 | 0,64 | 1,00 | 1,00 | 0,10 | 10,84 |
| *06:02 | 3 | 6 | 3,75 | 0,15 | 1,00 | 0,71 | 18,69 |
| *06:03 | 2 | 13 | 0,71 | 1,00 | 1,00 | 0,15 | 4,07 |
| *06:04 | 0 | 3 | 0,64 | 1,00 | 1,00 | 0,10 | 10,84 |
| ***HLA-DRB1*** | n=7 | n=36 |  |  |  |  |  |
| *01:01 | 1 | 2 | 2,83 | 0,42 | 1,00 | 0,36 | 28,48 |
| *01:02 | 0 | 6 | 0,31 | 0,57 | 1,00 | 0,06 | 5,17 |
| *03:01 | 3 | 7 | 3,11 | 0,32 | 1,00 | 0,61 | 15,31 |
| *04:01 | 0 | 1 | 1,58 | 1,00 | 1,00 | 0,18 | 27,96 |
| *04:03 | 0 | 1 | 1,58 | 1,00 | 1,00 | 0,18 | 27,96 |
| *04:04 | 0 | 3 | 0,64 | 1,00 | 1,00 | 0,10 | 10,84 |
| *07:01 | 2 | 7 | 1,66 | 0,62 | 1,00 | 0,33 | 9,75 |
| *08:01 | 0 | 7 | 0,26 | 0,32 | 1,00 | 0,05 | 4,32 |
| *10:01 | 0 | 2 | 0,92 | 1,00 | 1,00 | 0,13 | 15,91 |
| *11:01 | 2 | 7 | 1,66 | 0,62 | 1,00 | 0,33 | 9,75 |
| *11:04 | 1 | 1 | 5,83 | 0,30 | 1,00 | 0,49 | 60,97 |
| *13:01 | 2 | 13 | 0,71 | 1,00 | 1,00 | 0,15 | 4,07 |
| *13:02 | 0 | 3 | 0,64 | 1,00 | 1,00 | 0,10 | 10,84 |
| *15:01 | 3 | 7 | 3,11 | 0,32 | 1,00 | 0,61 | 15,31 |
| *15:02 | 0 | 3 | 0,64 | 1,00 | 1,00 | 0,10 | 10,84 |
| *16:01 | 0 | 2 | 0,92 | 1,00 | 1,00 | 0,13 | 15,91 |

## Supplementary Table 4. Association of HLA-I and HLA-II alleles with gastrointestinal involvement in *CTLA4* mutation carriers

| **Allele** | **Gastrointestinal involvement** | **No gastrointestinal involvement** | **Odds.Ratio** | **p.value** | **p.adjusted**  **(Bonferroni)** | **Lower.CI** | **Upper.CI** |
| --- | --- | --- | --- | --- | --- | --- | --- |
| ***HLA-A*** | n=22 | n=16 |  |  |  |  |  |
| *01:01 | 5 | 6 | 0,49 | 0,47 | 1,00 | 0,13 | 2,00 |
| *02:01 | 10 | 6 | 1,39 | 0,74 | 1,00 | 0,38 | 4,86 |
| *03:01 | 7 | 4 | 1,40 | 0,73 | 1,00 | 0,34 | 5,37 |
| *03:20 | 0 | 1 | 0,23 | 0,20 | 1,00 | 0,03 | 4,17 |
| *11:01 | 1 | 2 | 0,33 | 0,56 | 1,00 | 0,05 | 3,41 |
| *23:01 | 0 | 1 | 0,23 | 0,20 | 1,00 | 0,03 | 4,17 |
| *24:02 | 9 | 5 | 1,52 | 0,74 | 1,00 | 0,40 | 5,46 |
| *26:01 | 1 | 1 | 0,71 | 1,00 | 1,00 | 0,07 | 7,60 |
| *29:02 | 1 | 0 | 2,30 | 0,51 | 1,00 | 0,13 | 18,50 |
| *30:01 | 0 | 1 | 0,23 | 0,20 | 1,00 | 0,03 | 4,17 |
| *30:02 | 3 | 1 | 2,37 | 0,62 | 1,00 | 0,24 | 14,06 |
| *68:01 | 2 | 0 | 4,02 | 0,50 | 1,00 | 0,23 | 25,51 |
| *68:02 | 1 | 2 | 0,33 | 0,56 | 1,00 | 0,05 | 3,41 |
| ***HLA-B*** | n=22 | n=16 |  |  |  |  |  |
| *07:02 | 7 | 5 | 1,03 | 1,00 | 1,00 | 0,27 | 3,86 |
| *08:01 | 4 | 1 | 3,33 | 0,37 | 1,00 | 0,35 | 17,97 |
| *15:01 | 2 | 0 | 4,02 | 0,50 | 1,00 | 0,23 | 25,51 |
| *15:71 | 0 | 1 | 0,23 | 0,20 | 1,00 | 0,03 | 4,17 |
| *18:01 | 1 | 1 | 0,71 | 1,00 | 1,00 | 0,07 | 7,60 |
| *27:02 | 1 | 1 | 0,71 | 1,00 | 1,00 | 0,07 | 7,60 |
| *35:01 | 3 | 3 | 0,68 | 0,68 | 1,00 | 0,13 | 3,55 |
| *35:02 | 1 | 3 | 0,21 | 0,29 | 1,00 | 0,04 | 2,05 |
| *35:03 | 4 | 2 | 1,56 | 1,00 | 1,00 | 0,26 | 7,66 |
| *35:08 | 4 | 1 | 3,33 | 0,37 | 1,00 | 0,35 | 17,97 |
| *38:01 | 3 | 0 | 5,92 | 0,14 | 1,00 | 0,35 | 33,40 |
| *39:06 | 1 | 1 | 0,71 | 1,00 | 1,00 | 0,07 | 7,60 |
| *44:02 | 1 | 0 | 2,30 | 0,51 | 1,00 | 0,13 | 18,50 |
| *44:03 | 5 | 2 | 2,06 | 0,68 | 1,00 | 0,35 | 9,48 |
| *51:01 | 3 | 4 | 0,47 | 0,43 | 1,00 | 0,10 | 2,39 |
| *51:08 | 0 | 1 | 0,23 | 0,20 | 1,00 | 0,03 | 4,17 |
| *52:01 | 1 | 3 | 0,21 | 0,29 | 1,00 | 0,04 | 2,05 |
| *57:01 | 1 | 2 | 0,33 | 0,56 | 1,00 | 0,05 | 3,41 |
| ***HLA-C*** | n=22 | n=16 |  |  |  |  |  |
| *01:02 | 1 | 0 | 2,30 | 0,51 | 1,00 | 0,13 | 18,50 |
| *02:02 | 1 | 1 | 0,71 | 1,00 | 1,00 | 0,07 | 7,60 |
| *03:03 | 0 | 1 | 0,23 | 0,20 | 1,00 | 0,03 | 4,17 |
| *03:04 | 2 | 0 | 4,02 | 0,50 | 1,00 | 0,23 | 25,51 |
| *04:01 | 14 | 9 | 1,36 | 0,74 | 1,00 | 0,38 | 4,83 |
| *05:01 | 1 | 1 | 0,71 | 1,00 | 1,00 | 0,07 | 7,60 |
| *07:01 | 4 | 2 | 1,56 | 1,00 | 1,00 | 0,26 | 7,66 |
| *07:02 | 7 | 5 | 1,03 | 1,00 | 1,00 | 0,27 | 3,86 |
| *12:02 | 1 | 3 | 0,21 | 0,29 | 1,00 | 0,04 | 2,05 |
| *12:03 | 5 | 2 | 2,06 | 0,68 | 1,00 | 0,35 | 9,48 |
| *14:02 | 2 | 4 | 0,30 | 0,22 | 1,00 | 0,06 | 1,85 |
| *16:01 | 2 | 1 | 1,50 | 1,00 | 1,00 | 0,15 | 10,59 |
| *16:02 | 0 | 1 | 0,23 | 0,20 | 1,00 | 0,03 | 4,17 |
| ***HLA-DQB1*** | n=24 | n=19 |  |  |  |  |  |
| *02:01 | 7 | 3 | 2,20 | 0,47 | 1,00 | 0,48 | 8,49 |
| *02:02 | 4 | 2 | 1,70 | 0,68 | 1,00 | 0,29 | 8,18 |
| *03:01 | 6 | 6 | 0,72 | 0,74 | 1,00 | 0,20 | 2,66 |
| *03:02 | 3 | 1 | 2,57 | 0,62 | 1,00 | 0,27 | 15,00 |
| *03:03 | 1 | 2 | 0,37 | 0,58 | 1,00 | 0,05 | 3,71 |
| *04:02 | 4 | 3 | 1,07 | 1,00 | 1,00 | 0,22 | 4,83 |
| *05:01 | 5 | 7 | 0,45 | 0,31 | 1,00 | 0,13 | 1,74 |
| *05:02 | 1 | 1 | 0,78 | 1,00 | 1,00 | 0,08 | 8,20 |
| *06:01 | 0 | 3 | 0,10 | 0,04 | 0,43 | 0,02 | 1,66 |
| *06:02 | 5 | 4 | 0,99 | 1,00 | 1,00 | 0,24 | 3,99 |
| *06:03 | 11 | 4 | 3,17 | 0,12 | 1,00 | 0,79 | 10,89 |
| *06:04 | 1 | 2 | 0,37 | 0,58 | 1,00 | 0,05 | 3,71 |
| ***HLA-DRB1*** | n=24 | n=19 |  |  |  |  |  |
| *01:01 | 0 | 3 | 0,10 | 0,04 | 0,57 | 0,02 | 1,66 |
| *01:02 | 2 | 4 | 0,34 | 0,38 | 1,00 | 0,07 | 2,05 |
| *03:01 | 7 | 3 | 2,20 | 0,47 | 1,00 | 0,48 | 8,49 |
| *04:01 | 1 | 0 | 2,49 | 0,50 | 1,00 | 0,14 | 19,76 |
| *04:03 | 1 | 0 | 2,49 | 0,50 | 1,00 | 0,14 | 19,76 |
| *04:04 | 2 | 1 | 1,64 | 1,00 | 1,00 | 0,17 | 11,37 |
| *07:01 | 5 | 4 | 0,99 | 1,00 | 1,00 | 0,24 | 3,99 |
| *08:01 | 4 | 3 | 1,07 | 1,00 | 1,00 | 0,22 | 4,83 |
| *10:01 | 2 | 0 | 4,33 | 0,49 | 1,00 | 0,25 | 27,11 |
| *11:01 | 4 | 5 | 0,56 | 0,48 | 1,00 | 0,14 | 2,38 |
| *11:04 | 1 | 1 | 0,78 | 1,00 | 1,00 | 0,08 | 8,20 |
| *13:01 | 11 | 4 | 3,17 | 0,12 | 1,00 | 0,79 | 10,89 |
| *13:02 | 1 | 2 | 0,37 | 0,58 | 1,00 | 0,05 | 3,71 |
| *15:01 | 6 | 4 | 1,25 | 1,00 | 1,00 | 0,30 | 4,80 |
| *15:02 | 0 | 3 | 0,10 | 0,04 | 0,57 | 0,02 | 1,66 |
| *16:01 | 1 | 1 | 0,78 | 1,00 | 1,00 | 0,08 | 8,20 |

## Supplementary Table 5. Association of HLA-I and HLA-II alleles with non-malignant lymphoproliferation in *CTLA4* mutation carriers

| **Allele** | **Lymphoproliferation** | **No lymphoproliferation** | **Odds.Ratio** | **p.value** | **p.adjusted**  **(Bonferroni)** | **Lower.CI** | **Upper.CI** |
| --- | --- | --- | --- | --- | --- | --- | --- |
| ***HLA-A*** | n=13 | n=25 |  |  |  |  |  |
| *01:01 | 3 | 8 | 0,64 | 0,71 | 1,00 | 0,16 | 2,96 |
| *02:01 | 7 | 9 | 2,07 | 0,32 | 1,00 | 0,54 | 7,50 |
| *03:01 | 3 | 8 | 0,64 | 0,71 | 1,00 | 0,16 | 2,96 |
| *03:20 | 0 | 1 | 0,60 | 0,53 | 1,00 | 0,07 | 10,75 |
| *11:01 | 1 | 2 | 0,96 | 1,00 | 1,00 | 0,13 | 9,56 |
| *23:01 | 0 | 1 | 0,60 | 0,53 | 1,00 | 0,07 | 10,75 |
| *24:02 | 5 | 9 | 1,11 | 1,00 | 1,00 | 0,30 | 4,28 |
| *26:01 | 0 | 2 | 0,35 | 0,53 | 1,00 | 0,05 | 6,04 |
| *29:02 | 1 | 0 | 6,12 | 0,12 | 1,00 | 0,33 | 48,30 |
| *30:01 | 1 | 0 | 6,12 | 0,12 | 1,00 | 0,33 | 48,30 |
| *30:02 | 0 | 4 | 0,18 | 0,28 | 1,00 | 0,03 | 2,98 |
| *68:01 | 1 | 1 | 2 | 1,00 | 1,00 | 0,18 | 20,83 |
| *68:02 | 1 | 2 | 0,96 | 1,00 | 1,00 | 0,13 | 9,56 |
| ***HLA-B*** | n=13 | n=25 |  |  |  |  |  |
| *07:02 | 5 | 7 | 1,61 | 0,71 | 1,00 | 0,41 | 6,27 |
| *08:01 | 3 | 2 | 3,45 | 0,31 | 1,00 | 0,53 | 18,55 |
| *15:01 | 1 | 1 | 2 | 1,00 | 1,00 | 0,18 | 20,83 |
| *15:71 | 0 | 1 | 0,60 | 0,53 | 1,00 | 0,07 | 10,75 |
| *18:01 | 0 | 2 | 0,35 | 0,53 | 1,00 | 0,05 | 6,04 |
| *27:02 | 0 | 2 | 0,35 | 0,53 | 1,00 | 0,05 | 6,04 |
| *35:01 | 3 | 3 | 2,20 | 0,39 | 1,00 | 0,41 | 11,19 |
| *35:02 | 2 | 2 | 2,09 | 0,59 | 1,00 | 0,31 | 13,52 |
| *35:03 | 3 | 3 | 2,20 | 0,39 | 1,00 | 0,41 | 11,19 |
| *35:08 | 1 | 4 | 0,44 | 0,64 | 1,00 | 0,08 | 4,13 |
| *38:01 | 0 | 3 | 0,24 | 0,28 | 1,00 | 0,04 | 4,06 |
| *39:06 | 1 | 1 | 2 | 1,00 | 1,00 | 0,18 | 20,83 |
| *44:02 | 1 | 0 | 6,12 | 0,12 | 1,00 | 0,33 | 48,30 |
| *44:03 | 2 | 5 | 0,73 | 1,00 | 1,00 | 0,15 | 4,26 |
| *51:01 | 2 | 5 | 0,73 | 1,00 | 1,00 | 0,15 | 4,26 |
| *51:08 | 0 | 1 | 0,60 | 0,53 | 1,00 | 0,07 | 10,75 |
| *52:01 | 0 | 4 | 0,18 | 0,28 | 1,00 | 0,03 | 2,98 |
| *57:01 | 1 | 2 | 0,96 | 1,00 | 1,00 | 0,13 | 9,56 |
| ***HLA-C*** | n=13 | n=25 |  |  |  |  |  |
| *01:02 | 0 | 1 | 0,60 | 0,53 | 1,00 | 0,07 | 10,75 |
| *02:02 | 0 | 2 | 0,35 | 0,53 | 1,00 | 0,05 | 6,04 |
| *03:03 | 0 | 1 | 0,60 | 0,53 | 1,00 | 0,07 | 10,75 |
| *03:04 | 1 | 1 | 2 | 1,00 | 1,00 | 0,18 | 20,83 |
| *04:01 | 8 | 15 | 1,07 | 1,00 | 1,00 | 0,28 | 3,95 |
| *05:01 | 1 | 1 | 2 | 1,00 | 1,00 | 0,18 | 20,83 |
| *07:01 | 4 | 2 | 5,11 | 0,15 | 1,00 | 0,80 | 24,86 |
| *07:02 | 5 | 7 | 1,61 | 0,71 | 1,00 | 0,41 | 6,27 |
| *12:02 | 0 | 4 | 0,18 | 0,28 | 1,00 | 0,03 | 2,98 |
| *12:03 | 1 | 6 | 0,26 | 0,38 | 1,00 | 0,05 | 2,44 |
| *14:02 | 2 | 4 | 0,95 | 1,00 | 1,00 | 0,19 | 5,71 |
| *16:01 | 2 | 1 | 4,36 | 0,27 | 1,00 | 0,42 | 30,24 |
| *16:02 | 0 | 1 | 0,60 | 0,53 | 1,00 | 0,07 | 10,75 |
| ***HLA-DQB1*** | n=14 | n=29 |  |  |  |  |  |
| *02:01 | 3 | 7 | 0,86 | 1,00 | 1,00 | 0,21 | 3,91 |
| *02:02 | 1 | 5 | 0,37 | 0,65 | 1,00 | 0,07 | 3,39 |
| *03:01 | 3 | 9 | 0,61 | 0,72 | 1,00 | 0,16 | 2,72 |
| *03:02 | 1 | 3 | 0,67 | 1,00 | 1,00 | 0,11 | 6,35 |
| *03:03 | 1 | 2 | 1,04 | 1,00 | 1,00 | 0,15 | 10,25 |
| *04:02 | 2 | 5 | 0,80 | 1,00 | 1,00 | 0,17 | 4,61 |
| *05:01 | 5 | 7 | 1,75 | 0,48 | 1,00 | 0,46 | 6,61 |
| *05:02 | 1 | 1 | 2,15 | 1,00 | 1,00 | 0,20 | 22,22 |
| *06:01 | 0 | 3 | 0,26 | 0,29 | 1,00 | 0,05 | 4,40 |
| *06:02 | 5 | 4 | 3,47 | 0,12 | 1,00 | 0,77 | 14,01 |
| *06:03 | 6 | 9 | 1,67 | 0,51 | 1,00 | 0,46 | 5,93 |
| *06:04 | 0 | 3 | 0,26 | 0,29 | 1,00 | 0,05 | 4,40 |
| ***HLA-DRB1*** | n=14 | n=29 |  |  |  |  |  |
| *01:01 | 2 | 1 | 4,67 | 0,24 | 1,00 | 0,45 | 32,00 |
| *01:02 | 2 | 4 | 1,04 | 1,00 | 1,00 | 0,21 | 6,13 |
| *03:01 | 3 | 7 | 0,86 | 1,00 | 1,00 | 0,21 | 3,91 |
| *04:01 | 1 | 0 | 6,56 | 0,12 | 1,00 | 0,36 | 51,32 |
| *04:03 | 0 | 1 | 0,66 | 1,00 | 1,00 | 0,08 | 11,54 |
| *04:04 | 1 | 2 | 1,04 | 1,00 | 1,00 | 0,15 | 10,25 |
| *07:01 | 2 | 7 | 0,52 | 0,69 | 1,00 | 0,12 | 2,94 |
| *08:01 | 2 | 5 | 0,80 | 1,00 | 1,00 | 0,17 | 4,61 |
| *10:01 | 1 | 1 | 2,15 | 1,00 | 1,00 | 0,20 | 22,22 |
| *11:01 | 2 | 7 | 0,52 | 0,69 | 1,00 | 0,12 | 2,94 |
| *11:04 | 0 | 2 | 0,38 | 1,00 | 1,00 | 0,06 | 6,51 |
| *13:01 | 6 | 9 | 1,67 | 0,51 | 1,00 | 0,46 | 5,93 |
| *13:02+ | 0 | 3 | 0,26 | 0,29 | 1,00 | 0,05 | 4,40 |
| *15:01 | 5 | 5 | 2,67 | 0,25 | 1,00 | 0,64 | 10,45 |
| *15:02 | 0 | 3 | 0,26 | 0,29 | 1,00 | 0,05 | 4,40 |
| *16:01 | 1 | 1 | 2,15 | 1,00 | 1,00 | 0,20 | 22,22 |

## Supplementary Table 6. Association of HLA-I and HLA-II alleles with cytopenia in *CTLA4* mutation carriers

| **Allele** | **Cytopenia** | **No cytopenia** | **Odds.Ratio** | **p.value** | **p.adjusted**  **(Bonferroni)** | **Lower.CI** | **Upper.CI** |
| --- | --- | --- | --- | --- | --- | --- | --- |
| ***HLA-A*** | n=5 | n=33 |  |  |  |  |  |
| *01:01 | 0 | 11 | 0,18 | 0,15 | 1,00 | 0,03 | 2,97 |
| *02:01 | 3 | 13 | 2,31 | 0,63 | 1,00 | 0,36 | 12,39 |
| *03:01 | 1 | 10 | 0,58 | 1,00 | 1,00 | 0,10 | 5,44 |
| *03:20 | 0 | 1 | 1,97 | 1,00 | 1,00 | 0,21 | 35,33 |
| *11:01 | 0 | 3 | 0,79 | 1,00 | 1,00 | 0,12 | 13,67 |
| *23:01 | 0 | 1 | 1,97 | 1,00 | 1,00 | 0,21 | 35,33 |
| *24:02 | 2 | 12 | 1,17 | 1,00 | 1,00 | 0,21 | 7,19 |
| *26:01 | 0 | 2 | 1,15 | 1,00 | 1,00 | 0,16 | 20,10 |
| *29:02 | 1 | 0 | 22,33 | 0,02 | 0,25 | 1,03 | 179,03 |
| *30:01 | 1 | 0 | 22,33 | 0,02 | 0,25 | 1,03 | 179,03 |
| *30:02 | 0 | 4 | 0,60 | 1,00 | 1,00 | 0,10 | 10,17 |
| *68:01 | 0 | 2 | 1,15 | 1,00 | 1,00 | 0,16 | 20,10 |
| *68:02 | 0 | 3 | 0,79 | 1,00 | 1,00 | 0,12 | 13,67 |
| ***HLA-B*** | n=5 | n=33 |  |  |  |  |  |
| *07:02 | 2 | 10 | 1,53 | 0,64 | 1,00 | 0,27 | 9,47 |
| *08:01 | 2 | 3 | 6,67 | 0,12 | 1,00 | 0,87 | 44,37 |
| *15:01 | 0 | 2 | 1,15 | 1,00 | 1,00 | 0,16 | 20,10 |
| *15:71 | 0 | 1 | 1,97 | 1,00 | 1,00 | 0,21 | 35,33 |
| *18:01 | 0 | 2 | 1,15 | 1,00 | 1,00 | 0,16 | 20,10 |
| *27:02 | 0 | 2 | 1,15 | 1,00 | 1,00 | 0,16 | 20,10 |
| *35:01 | 1 | 5 | 1,40 | 1,00 | 1,00 | 0,22 | 13,57 |
| *35:02 | 1 | 3 | 2,50 | 0,45 | 1,00 | 0,34 | 25,03 |
| *35:03 | 1 | 5 | 1,40 | 1,00 | 1,00 | 0,22 | 13,57 |
| *35:08 | 0 | 5 | 0,47 | 0,57 | 1,00 | 0,08 | 7,97 |
| *38:01 | 0 | 3 | 0,79 | 1,00 | 1,00 | 0,12 | 13,67 |
| *39:06 | 0 | 2 | 1,15 | 1,00 | 1,00 | 0,16 | 20,10 |
| *44:02 | 1 | 0 | 22,33 | 0,02 | 0,35 | 1,03 | 179,03 |
| *44:03 | 1 | 6 | 1,13 | 1,00 | 1,00 | 0,18 | 10,80 |
| *51:01 | 1 | 6 | 1,13 | 1,00 | 1,00 | 0,18 | 10,80 |
| *51:08 | 0 | 1 | 1,97 | 1,00 | 1,00 | 0,21 | 35,33 |
| *52:01 | 0 | 4 | 0,60 | 1,00 | 1,00 | 0,10 | 10,17 |
| *57:01 | 0 | 3 | 0,79 | 1,00 | 1,00 | 0,12 | 13,67 |
| ***HLA-C*** | n=5 | n=33 |  |  |  |  |  |
| *01:02 | 0 | 1 | 1,97 | 1,00 | 1,00 | 0,21 | 35,33 |
| *02:02 | 0 | 2 | 1,15 | 1,00 | 1,00 | 0,16 | 20,10 |
| *03:03 | 0 | 1 | 1,97 | 1,00 | 1,00 | 0,21 | 35,33 |
| *03:04 | 0 | 2 | 1,15 | 1,00 | 1,00 | 0,16 | 20,10 |
| *04:01 | 3 | 20 | 0,98 | 1,00 | 1,00 | 0,16 | 5,37 |
| *05:01 | 1 | 1 | 8 | 0,25 | 1,00 | 0,61 | 85,26 |
| *07:01 | 2 | 4 | 4,83 | 0,17 | 1,00 | 0,70 | 31,37 |
| *07:02 | 2 | 10 | 1,53 | 0,64 | 1,00 | 0,27 | 9,47 |
| *12:02 | 0 | 4 | 0,60 | 1,00 | 1,00 | 0,10 | 10,17 |
| *12:03 | 0 | 7 | 0,32 | 0,32 | 1,00 | 0,06 | 5,39 |
| *14:02 | 1 | 5 | 1,40 | 1,00 | 1,00 | 0,22 | 13,57 |
| *16:01 | 1 | 2 | 3,88 | 0,35 | 1,00 | 0,44 | 39,94 |
| *16:02 | 0 | 1 | 1,97 | 1,00 | 1,00 | 0,21 | 35,33 |
| ***HLA-DQB1*** | n=7 | n=36 |  |  |  |  |  |
| *02:01 | 1 | 9 | 0,50 | 1,00 | 1,00 | 0,10 | 4,57 |
| *02:02 | 1 | 5 | 1,03 | 1,00 | 1,00 | 0,18 | 9,70 |
| *03:01 | 2 | 10 | 1,04 | 1,00 | 1,00 | 0,22 | 6,02 |
| *03:02 | 2 | 2 | 6,80 | 0,12 | 1,00 | 0,87 | 45,00 |
| *03:03 | 0 | 3 | 0,64 | 1,00 | 1,00 | 0,10 | 10,84 |
| *04:02 | 0 | 7 | 0,26 | 0,32 | 1,00 | 0,05 | 4,32 |
| *05:01 | 2 | 10 | 1,04 | 1,00 | 1,00 | 0,22 | 6,02 |
| *05:02 | 0 | 2 | 0,92 | 1,00 | 1,00 | 0,13 | 15,91 |
| *06:01 | 0 | 3 | 0,64 | 1,00 | 1,00 | 0,10 | 10,84 |
| *06:02 | 3 | 6 | 3,75 | 0,15 | 1,00 | 0,71 | 18,69 |
| *06:03 | 2 | 13 | 0,71 | 1,00 | 1,00 | 0,15 | 4,07 |
| *06:04 | 1 | 2 | 2,83 | 0,42 | 1,00 | 0,36 | 28,48 |
| ***HLA-DRB1*** | n=7 | n=36 |  |  |  |  |  |
| *01:01 | 1 | 2 | 2,83 | 0,42 | 1,00 | 0,36 | 28,48 |
| *01:02 | 1 | 5 | 1,03 | 1,00 | 1,00 | 0,18 | 9,70 |
| *03:01 | 1 | 9 | 0,50 | 1,00 | 1,00 | 0,10 | 4,57 |
| *04:01 | 1 | 0 | 16,85 | 0,03 | 0,47 | 0,84 | 133,07 |
| *04:03 | 0 | 1 | 1,58 | 1,00 | 1,00 | 0,18 | 27,96 |
| *04:04 | 2 | 1 | 14 | 0,06 | 1,00 | 1,17 | 98,51 |
| *07:01 | 1 | 8 | 0,58 | 1,00 | 1,00 | 0,11 | 5,35 |
| *08:01 | 0 | 7 | 0,26 | 0,32 | 1,00 | 0,05 | 4,32 |
| *10:01 | 0 | 2 | 0,92 | 1,00 | 1,00 | 0,13 | 15,91 |
| *11:01 | 1 | 8 | 0,58 | 1,00 | 1,00 | 0,11 | 5,35 |
| *11:04 | 0 | 2 | 0,92 | 1,00 | 1,00 | 0,13 | 15,91 |
| *13:01 | 2 | 13 | 0,71 | 1,00 | 1,00 | 0,15 | 4,07 |
| *13:02 | 1 | 2 | 2,83 | 0,42 | 1,00 | 0,36 | 28,48 |
| *15:01 | 3 | 7 | 3,11 | 0,32 | 1,00 | 0,61 | 15,31 |
| *15:02 | 0 | 3 | 0,64 | 1,00 | 1,00 | 0,10 | 10,84 |
| *16:01 | 0 | 2 | 0,92 | 1,00 | 1,00 | 0,13 | 15,91 |
